# Parameter-Free Determination of Au Nanorod Dimensions Using Depolarized DLS and Genetic Optimization

**DOI:** 10.1021/acs.jpcb.5c06410

**Published:** 2026-01-29

**Authors:** Nehal Nupnar, Geofrey Nyabere, Claire M. B. Bolding, Kiril A. Streletzky, Michael J. A. Hore

**Affiliations:** † Department of Macromolecular Science and Engineering, 2546 Case Western Reserve University, 10900 Euclid Avenue, Cleveland, Ohio 44106, United States; ‡ Department of Physics, 2564 Cleveland State University, 2121 Euclid Avenue, Cleveland, Ohio 44115, United States

## Abstract

Gold nanorods (AuNRs) have received considerable attention for their distinctive optical properties and well-defined, low-polydispersity dimensions. These characteristics position them as promising candidates for diverse applications in imaging, sensing, and treating diseases. However, accurate characterization of AuNRs in their native solution state, which is crucial to many applications, presents many challenges—especially if AuNRs are coated with surface layers (e.g., surfactants or grafted polymers). When applied to AuNRs with functionalized surfaces, common techniques such as transmission electron microscopy (TEM), small-angle scattering, and dynamic light scattering (DLS) can present limitations such as small sample sizes, the inability to detect light elements, a lack of a comprehensive analytical framework, and/or a dependence on *a priori* information about the particle dimensions. In this work, we focus on multiangle depolarized DLS (DDLS) measurements of three distinct, surfactant-coated AuNRs samples in solution. DDLS data was analyzed using two analytical approaches and compared with a genetic algorithm analysis that optimizes the dimensions of the particles to best match relaxation rates obtained from DDLS. For samples that produced high-quality DDLS data, all three approaches yielded length estimates that were highly consistent (within 10–20%) with dimensions obtained from TEM/SEM. In contrast, noisy DDLS data posed challenges for direct analysis, and the genetic algorithm approach emerged as particularly advantageous, providing dimensions that more closely aligned with TEM/SEM values than the analytical methods. Our results suggest that the genetic algorithm can accurately capture the dimensions of the AuNRs from their rotational and translational relaxation rates alone, without the need for additional information (e.g., aspect ratio). Looking to the future, this approach to analyzing DDLS measurements will allow the technique to capture important structural information on more complex, anisotropic nanoparticle systems to enable their use in a wide range of applications.

## Graphical abstract

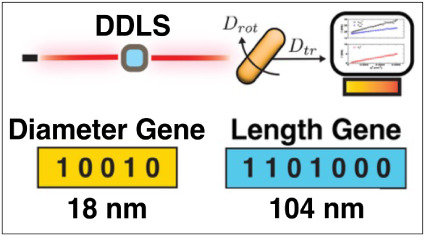

## Introduction

Polymer nanocomposites play a pivotal role in advanced material technologies because of the unique combination of physical properties that appear when a polymer matrix is combined with nanoparticles. The blending of nanoparticles with a polymer matrix can result in improved conductivity, mechanical strength, optical characteristics, and other properties. Gold nanorods (AuNRs), particularly when functionalized with polymer grafts, can impart tunable optical extinction and polarization to nanocomposite materials, making them ideal candidates for applications ranging from surface coatings to sensing technologies.^1–5^

To purposefully design nanocomposite materials that exploit the physical properties of nanorods, it is important to be able to characterize the size, aspect ratio (ν = length/diameter), and transport characteristics (e.g., translational and rotational diffusion coefficients) of the nanoparticles. Such characterizations can inform nanocomposite processing conditions, provide mechanistic insight into behaviors of the materials, and lead to predictions, for example, of the optical extinction of a nanocomposite. In addition, accurate characterization of nanorods is key to understanding the structure and dynamics of polymers that may be grafted to the nanorod surface. Bare nanorods can be analyzed using a variety of techniques. For instance, determining the aspect ratio of AuNRs often entails utilizing the optical absorption spectra of the metallic nanorods themselves, specifically using the wavelength of the longitudinal surface plasmon resonance (LSPR).^6,7^ However, an ongoing debate persists regarding the relationship between the theoretically expected and experimentally observed absorption spectra for metallic nanorods. Recent theoretical and experimental studies suggest that contrary to common assumptions, the geometrical aspect ratio of the nanorods does not singularly dictate the absorption peak of the surface plasmon resonance.^6–10^ In addition, any dispersity in the sizes and shapes of an ensemble of Au nanorods, including the presence of spherical impurities, cannot be directly quantified through the optical absorption spectrum. As an alternative to spectroscopic techniques, electron microscopy (EM) allows for the direct measurement of particle size distributions and self-assembled structures. However, even with automation and machine learning-directed measurements,^10–12^ EM is a relatively low-throughput technique that can only sample a limited number of particles. Moreover, EM offers limited insights into polymer-grafted systems and typically does not provide solution-state information without specialized sample cells.^13^ Finally, both electron microscopy and optical spectroscopy fail to offer detailed insights into the behavior of organic molecules (e.g., polymers or surfactants) grafted to the nanorod surface, such as their size/thickness, conformation, or location on the surface.

Information regarding the behavior of surface layers can be obtained, in principle, with reliable measurements of bare nanorod dimensions and/or diffusion coefficients in solution. Small-angle neutron scattering (SANS), for example, has the potential to characterize both AuNR dimensions and surface layer properties^14^ but has yet to be used to fully characterize polymer-grafted AuNRs due to the lack of analytical, single-particle form factors. Mukhopadhyay and coworkers^15,16^ have made measurements of AuNR diffusion in complex fluid environments such as those containing spherical nanoparticles or bottlebrush polymers using two-photon fluctuation correlation spectroscopy (FCS). While this method is powerful, it can also be more costly and challenging to use when compared to dynamic light scattering (DLS)—especially in cases where identifying a particular location of the particles, such as within a living cell, is not necessary. DLS has been used to provide insight into surface layers on spherical nanoparticles,^17^ but accurately characterizing polymer-grafted, geometrically anisotropic nanoparticles with DLS remains challenging due to the nonspherical geometry of the nanoparticles. Nevertheless, DLS can be powerful for fast characterization of a dilute solution of uniform particles, enabling the determination of hydrodynamic particle sizes within their natural environment. Traditional DLS produces a hydrodynamic size under the assumption that the particles are spherical and monodisperse. By adding a polarizer and an analyzer to a DLS instrument (i.e., depolarized DLS, DDLS), the technique is able to separately resolve the translational (*D*_tr_) and rotational (*D*_rot_) diffusion coefficients of geometrically anisotropic particles, offering a complementary approach to DLS for analyzing nonspherical nanoparticles in addition to providing a particle’s rotational dynamics.

DLS operates by measuring the intensity-intensity autocorrelation function *g* ^(2)^(*q*,τ), which is used to obtain the field-field autocorrelation function *g* ^(1)^(*q*,τ) through the Siegert relation. The Brownian motion of nanoparticles gives rise to intensity fluctuations of scattered light that can be analyzed using *g* ^(1)^(*q*,τ) and depends strongly on *D*_tr_ of nanoparticles. The translational diffusion coefficient obtained from *g* ^(1)^(*q*,τ) is used to estimate the hydrodynamic size of a particle in solution, via the Stokes–Einstein equation.^18^ More advanced analysis techniques based on regularization algorithms can also be used to invert the particle size distribution for irregular particle shapes, but such methods produce a single representative particle size rather than providing detailed particle dimensions.^19^ For geometrically anisotropic particles like the tobacco mosaic virus,^20,21^ AuNRs,^22^ carbon nanotubes,^23^ three-armed star elastin-like polypeptides,^24^ supramolecular hydrogels,^25^ and cellulose nanocrystals,^26^ both *D*_tr_ and *D*_rot_ strongly influence the decay of the *g* ^(1)^(*q*,τ). These influences can be separated by studying both the polarized (VV) and depolarized (VH) autocorrelation functions of the scattering signal. By measuring both the polarized and depolarized signals and analyzing their angular dependence, it becomes feasible to extract the actual dimensions of geometrically anisotropic particles like AuNRs. DDLS is not only useful in determining the length, diameter, and aspect ratio of rods but can also provide information on polydispersity and solubility of the rods.^8,27^

The analysis of DDLS measurements is based on several assumptions that have yet to undergo comprehensive testing across a spectrum of sizes, shapes, and materials. Predictions of *D*_tr_ and *D*_rot_ typically rely on shape functions that are limited to special cases yielding analytical solutions (e.g., prolate ellipsoids).^28^ To analyze more complicated geometries of particles or polymer-grafted systems, the appropriate shape functions must be determined first. Furthermore, DDLS measurements are complicated as a depolarized signal is often weak in comparison to a polarized signal. Additionally, the contributions of translational and rotational Brownian motion to the decay of *g* ^(1)^(*q*,τ) can be difficult to separate in certain cases. Early work from Rodriguez et al.^29^ demonstrated that bare AuNRs can be characterized with DDLS. Glidden et al.^8^ developed an in-house DDLS instrument that employed time-tagged time-resolved (TTTR) photon collection, which facilitated measurements of *D*_tr_ and *D*_rot_ at a single scattering angle. Analysis of their DDLS measurements yielded AuNR dimensions that were accurate to within 10–20% of the actual sizes but required *a priori* knowledge of the particles’ aspect ratio. In their measurements, the authors noted a low count rate of scattered photons and did not have the opportunity to analyze the angular dependence of scattering from AuNRs. Bryant et al.^27^ similarly performed DDLS measurements of AuNRs in suspension and discovered that the translational diffusion motion can be uniquely distinguished from the VV scattering autocorrelation function by looking at the *q* ^2^τ scaling of *g* ^(1)^(*q*,τ). Their analysis took into account measurements from different angles. However, for some rod sizes, the extracted diffusion coefficients differed from theoretical predictions by more than 40%. For instance, *D*_tr_ for AuNRs with ν = 8.1 was underestimated by the Broersma model^30^ by ∼41%, while *D*_rot_ for AuNRs with ν = 2.9 was underestimated by ∼40%.

Multiangle DDLS shows significant potential as a technique for characterizing anisotropic nanoparticles, such as AuNRs, directly in solution. It is easier and cheaper than EM methods, FCS, SANS/SAXS, and AFM. It has an additional advantage of probing a large number of particles. However, DDLS requires careful attention to address low count rate VH signals, higher-order contributions of rotational motion to VV signals, and the effects of the sample polydispersity—all of which can complicate quantitative interpretation and motivate the need for new and robust analysis approaches. Although purification techniques like asymmetrical field-flow fractionation^31^ have been used to enhance the uniformity of AuNRs samples, advances in AuNRs synthesis protocols^32,33^ have led to greater sample purity (e.g., a significant reduction in spherical impurities) and improved tunability of AuNR dimensions.

Here, we performed multiangle DDLS on three commercial AuNR samples with aspect ratios between 3.5 and 6. We analyzed the measurements using three distinct approaches which aimed for precise and rapid characterization of nanorods within their natural solution environment using no prior information (e.g., without requiring dimensions from other techniques or sources). We demonstrate that even in cases where the AuNR samples were of relatively poor quality or we were provided with incorrect specifications, DDLS was able to quickly determine accurate particle dimensions. We analyzed our DDLS measurements using three geometrical models: a straight cylinder (SC), prolate ellipsoid (PE), and a spherocylinder (SP). Two approaches relied on either directly calculating or guessing a representative aspect ratio to obtain a direct match to the particles’ translational and rotational diffusion coefficients, while a third approach using a genetic optimization algorithm evolved initial values of particle dimensions until they best matched the translational and rotational relaxation rates at all angles. Our estimated dimensions were generally in agreement with values obtained from EM and showed slightly larger dimensions that arise presumably from the presence of surfactant on the nanoparticle surface. Our analysis helps establish a foundation for the subsequent characterization of polymer-grafted nanorods and other more complex particle geometries in the future, especially for instances in which postsynthetic modification of nanoparticles alters their dimensions and aspect ratio.

## Experimental Methods

### Gold Nanorod Sample Preparation

Gold nanorods (AuNRs) with specific nominal dimensions were obtained from Nanopartz, Inc. (Loveland, Colorado, USA) as colloidal dispersions in deionized (DI) water. We designated our samples as AuNRs XXX, where XXX is the wavelength (in nm) of the longitudinal surface plasmon resonance (LSPR) of the particles. Three samples were chosen which, according to manufacturer specifications, had identical diameters (*D*) and differed only in their average length (*L*): AuNRs 980 (*D* × *L* = 10 × 59 nm), AuNRs 900 (10 × 50 nm), and AuNRs 750 (10 × 35 nm). The AuNRs’ dimensions were validated by transmission electron microscopy (TEM) and scanning electron microscopy (SEM). As will be discussed, we observed significant differences between the dimensions reported by the manufacturer and those observed from electron microscopy. Purchased AuNRs were coated with a cetyltrimethylammonium bromide (CTAB) bilayer. Based on previously published small-angle neutron scattering (SANS) measurements^14^ on similar nanorods, the CTAB layer has a thickness of 3.6 nm. In preparation for dynamic light scattering measurements, stock solutions of AuNRs were directly passed through poly­(ether sulfone) (PES) filters (pore size 0.2 μm) into scintillation vials. The concentrations of the AuNR dispersions used for light scattering measurements were 0.042 mg/mL (AuNRs 980 and AuNRs 750) and 0.035 mg/mL (AuNRs 900). These concentrations were selected to maximize the scattering signal while remaining low enough to minimize multiple scattering and interparticle interactions. The filtered solutions were placed in an ultrasonic water bath for 30 min to eliminate trapped air, and 2 mL aliquots were passed through a second PES filter into precleaned borosilicate glass light scattering cells.

### Transmission Electron Microscopy

Transmission electron microscopy (TEM) was used to image three samples of AuNRs to measure their dimensions. TEM samples were prepared by diluting AuNRs to a mass fraction of 0.01% in H_2_O, placing them in an ultrasonic bath for 10 min, and subsequently drop casting onto a TEM grid. Carbon film TEM grids were plasma-treated for 30 s using the PELCO easiGlow Glow Discharge Cleaning System. Finally, 6 μL of the sample dispersion was dropped onto a grid and allowed to absorb for 5 min before any excess water was wicked away. The grid was then transferred to a single-tilt sample holder and imaged on an FEI Tecnai 20 TEM operating at 200 kV.

### Scanning Electron Microscopy

Scanning electron microscopy (SEM) images of all three nanorods were acquired using an FEI Inspect F50 operating at 20 kV with a dwell time of 30 μs. Samples for SEM imaging were prepared using the protocol for TEM samples, except a 1 μL drop of AuNRs was cast onto a plasma-etched silicon substrate. The silicon substrate was affixed to an SEM sample stub using carbon tape, with a small drop of silver epoxy on the corner serving as a conductive path. A brief sputtering of 30 s was employed to further reduce charging effects.

### UV–Visible Spectroscopy (UV–Vis)

Optical absorbance measurements were made with a Genesys 10S UV spectrometer (Thermo Fisher) in disposable plastic cuvettes. AuNRs solutions were measured as-received to validate the reported LSPR wavelengths. UV–vis measurement readings were corrected to eliminate any background contributions from the solvent or instrumentation.

### Depolarized Dynamic Light Scattering (DDLS)

Depolarized dynamic light scattering (DDLS) was performed using a modified dynamic light scattering instrument (Brookhaven Instruments, BI-200SM) with a diode laser with wavelength λ = 637 nm and power of 40 mW. We modified the optical design following refs.^18^ and^24^ to enhance the accuracy and sensitivity of the measurements. A Glan-Thompson calcite polarizer (Thorlabs Inc., GTH5M-A) was positioned directly in front of the laser source to ensure that only vertically polarized light impinged upon the sample. A Glan-Thompson analyzer module (Brookhaven Instruments, BI-PA) with a two-position, click-stop stage was placed in the optical pathway preceding the detector to capture vertically (VV) and horizontally (VH) polarized scattered signals. The measurements were conducted at room temperature (*T* ≈ 25 °C) and at scattering angles ranging from 30° to 140° with a step size of 5°, enabling a comprehensive analysis of the nanorods’ translational and rotational motion. Figure 1 illustrates the setup of our DDLS instrument as viewed from the top.

**Figure 1:**
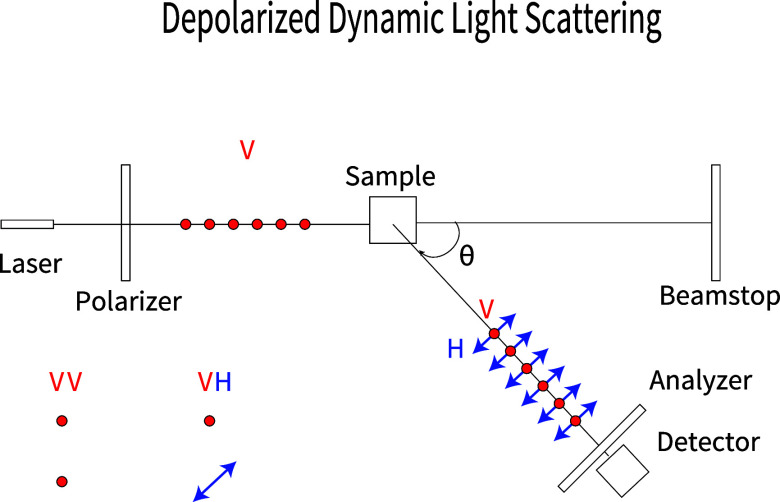
Schematic of the DDLS instrument as viewed from the top. Laser light is passed through a polarizer prior to the sample and an analyzer after the sample. Scattering intensities are collected at various scattering angles θ, with the analyzer in the same position as the polarizer (VV measurement) or rotated 90° from the orientation of the polarizer (VH measurement).

The analyzer position was maintained vertically to collect a VV signal. The VV scattered signals lie in the plane perpendicular to the plane of the page, as shown by the red dots in Figure 1. To collect VH signals, the analyzer position was maintained horizontally. The VH scattered signals lie in the plane of the page, as shown by the blue double-headed arrows in Figure 1. Both vertically (VV) and horizontally (VH) polarized signals were measured by the detector utilizing a pinhole of 100 nm for AuNRs 980 and a pinhole of 200 nm for AuNRs 900 and AuNRs 750. The light scattering data were collected over 3 min for AuNRs 980 and 2 min for AuNRs 900 and AuNRs 750.

DDLS directly measured VV and VH intensity–intensity autocorrelation functions $g_{VV}^{\left(2\right)}\left(q,\tau \right)$ and $g_{VH}^{\left(2\right)}\left(q,\tau \right)$ at various scattering angles. The analysis of the autocorrelation functions and interpretation of the DDLS scattering by rod-like monodisperse particles in dilute solutions^34,35^ depend on the relative length *L* of the particles in comparison to the magnitude of the light scattering vector, *q*, defined as

1 $$q=\left(\frac{4\pi n}{\lambda }\right)\sin\left(\frac{\theta }{2}\right)$$

Here, *n* represents the refractive index of the solution, λ is the wavelength of the incident light in a vacuum, and θ is the scattering angle between the incident and scattered light. In this work, *q* ranged from 0.0068 to 0.025 nm^–1^, corresponding to probed length scales (*d* = 2π/*q*) of approximately 924 to 251 nm, respectively.

For spheres and short rods of length *L* such that *qL* < 3–5, the VV signal is expected to contain information only about their translational motion. Consequently, $g_{VV}^{\left(2\right)}\left(q,\tau \right)$ should depend only on the translational relaxation rate, Γ_tr_:

2 $$g_{VV}^{\left(2\right)}\left(q,\tau \right)=A_{1}\exp\left(-2\Gamma_{tr}\tau \right)+B$$

where *A*_1_ represents the amplitude of the decay function and *B* is a baseline corresponding to $g_{VV}^{\left(2\right)}\left(q,\tau \to \infty \right)$ . If *L* is such that 5 < *qL* < 10 (intermediate length regime), then the VV autocorrelation function is expected to be bimodal and contain contributions from both the translational and rotational motions of the nanorods. In this case,

3 $$g_{VV}^{\left(2\right)}\left(q,\tau \right)={\{A_{1}\exp\left(-\Gamma_{tr}\tau \right)+A_{2}\exp\left(-\Gamma_{\mathrm{mix}}\tau \right)\}}^{2}+B$$

where Γ_mix_ = Γ_tr_ + Γ_rot_, Γ_rot_ is the rotational relaxation rate, *A*_2_ is the amplitude of the second decay, and the amplitudes obey the constraint *A*_1_ + *A*_2_ = 1. The VH intensity–intensity autocorrelation function $g_{VH}^{\left(2\right)}$ for both the short (*qL* < 3) and intermediate (5 < *qL* < 10) length regimes depends on Γ_tr_ and Γ_rot_. As a result, $g_{VH}^{\left(2\right)}\left(q,\tau \right)$ is expressed as

4 $$g_{VH}^{\left(2\right)}\left(q,\tau \right)=A\exp\left(-2\Gamma_{\mathrm{mix}}\tau \right)+B$$

with *B* representing the baseline $g_{VH}^{\left(2\right)}\left(q,\tau \to \infty \right)$ . For analysis, both the VV and VH autocorrelation functions are recast as normalized functions $g_{VV}^{\left(2\right)}{\left(q,\tau \right)}^{*}$ and $g_{VH}^{\left(2\right)}{\left(q,\tau \right)}^{*}$ using the following equation:

5 $$g^{\left(2\right)}{\left(q,\tau \right)}^{*}=\frac{g^{\left(2\right)}\left(q,\tau \right)-B}{g^{\left(2\right)}\left(q,\tau_{\min}\right)-B}$$

For simplicity, we will refer to the normalized functions as the autocorrelation functions throughout the remainder of this article. Since $g_{VV}^{\left(2\right)}\left(q,\tau \right)$ and $g_{VH}^{\left(2\right)}\left(q,\tau \right)$ were measured independently, we will refer to mixed relaxation rates taken from the fast VV mode and VH as $\Gamma_{\mathrm{mix}}^{a}$ and $\Gamma_{\mathrm{mix}}^{b}$ , respectively. The relaxation rates observed experimentally are related to the diffusion coefficients of the associated motions, i.e.:

6 $$\Gamma_{tr}=D_{tr}q^{2}$$

7 $$\Gamma_{\mathrm{rot}}=6D_{\mathrm{rot}}$$

where *D*_tr_ and *D*_rot_ are the translational and rotational diffusion coefficients.

The autocorrelation functions can be plotted as a function of *q* ^2^τ. As a result, the autocorrelation functions containing contributions from translational diffusion will superimpose in this representation (cf. eq 2). For instances in which multiple decays are observed (e.g., the intermediate length regime described by eq 3), only the decay corresponding to translational diffusion will superimpose.^27^ Thus, by observing the scaling of the bimodal autocorrelation functions with *q* ^2^, it is possible to identify a particular mode and a range of scattering angles where observed decays can be attributed solely to translational diffusion. In contrast to the translational diffusion coefficient, the rotational diffusion coefficient provides a constant contribution at all scattering angles, and the corresponding autocorrelation functions would appear separated when plotted as a function of *q* ^2^τ.

### Genetic Optimization Algorithm

The task of determining the dimensions of anisotropic nanoparticles from DDLS measurements can be viewed as an optimization problem, where the values of *D* (particle diameter) and *L* (particle length) are identified such that the calculated relaxation rates best match those measured experimentally. Given our relatively small parameter space of *L* and *D*, a grid search through all possible combinations of these values would be an effective method for analyzing the DDLS measurements. However, as samples become more complex (e.g., by grafting them with polymers), a grid search is expected to be less efficient than a genetic optimization algorithm (GA). Using an optimization-based approach is particularly beneficial, as natural dispersities in the dimensions of nanorods can lead to differences between theoretical predictions of Γ_tr_ and Γ_rot_, and those observed by DDLS. In this study, we used a GA to assist in the interpretation of DDLS measurements, with the motivation of quickly determining AuNRs dimensions without the need for any *a priori* knowledge of the particles (e.g., manufacturer’s specifications, microscopy images, plasmon resonances, etc.) with an eye toward applying the GA approach to more complex systems in the future. Recently, Wu and coworkers^36^ used a two-stage process involving a neural network and GA to interpret DDLS measurements. In their approach, a random vector functional link neural network (RVFLNN) was trained to fit VV and VH autocorrelation functions to obtain Γ_tr_ and Γ_mix_. The authors used these relaxation rates to compute *D*_tr_ and *D*_rot_, and a GA was used to optimize choices of *L* and *D* to closely match the diffusion coefficients. In contrast, our GA seeks to optimize choices of *L* and *D* to simultaneously match the relaxation rates at all angles, providing the algorithm with an expanded collection of values to optimize against.

We illustrate our GA schematically in Figure 2. The GA encodes potential solutions to the nanorod characterization problem as binary numbers that represent the average dimensions of the nanoparticles (i.e., *L* and *D*). Each bit of the dimensions corresponds to a “gene” that can be mutated by flipping its value. Genes are randomly selected for mutation and flipped with a probability *p*_*M*_. Similarly, groups of sequential genes, having random offsets from the most significant bit and random ranges/lengths, are modified through crossover operations in which the groups of genes between two independent solutions are swapped with a probability *p*_*C*_.

**Figure 2:**
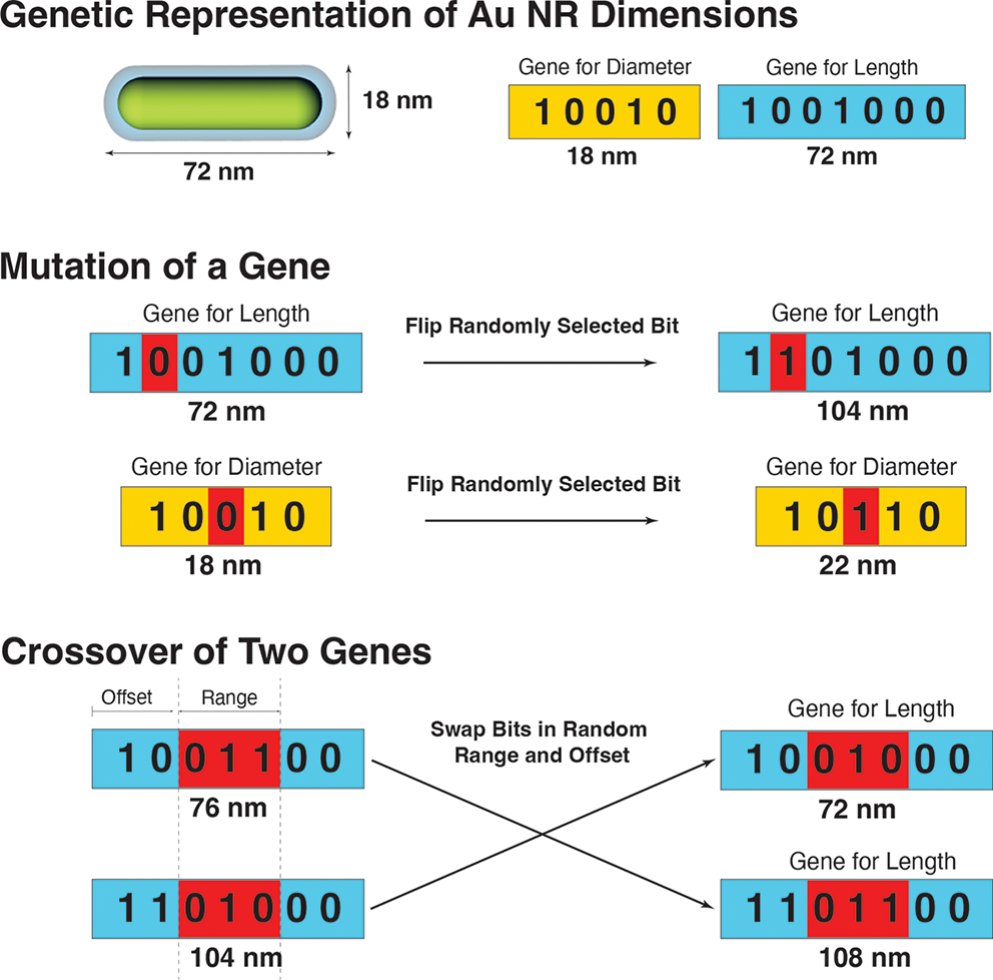
Schematic of the genetic optimization algorithm. The length (*L*) and diameter (*D*) of the nanorod are separately encoded as two binary numbers where each bit represents a gene. Optimization of the nanorod dimensions is performed through two genetic processes: Mutation of a gene caused by randomly inverting its value, and crossover of genes, which occurs by swapping two regions of random length between two independent sets of genes.

Our implementation starts with a population of *N*_*p*_ = 100 randomly generated pairs of (*L*, *D*) that represent potential solutions to the DDLS optimization problem. To determine how well each solution describes the experimentally determined relaxation rates, we compute a fitness function based on the squared sum of errors (SSE) between the theoretical and measured relaxation rates at each value of *q*. For a particular individual *i* in the population, the SSE is defined as

8 $$SSE_{i}\left(\Gamma_{tr},\Gamma_{\mathrm{rot}}\right)=\underset{q}{\sum }{\left(\Gamma_{tr,q}-\Gamma_{tr,q}^{0}\right)}^{2}+\underset{q}{\sum }{\left(\Gamma_{\mathrm{rot},q}-\Gamma_{\mathrm{rot},q}^{0}\right)}^{2}$$

with the subscript *q* indicating the scattering vector to which the values correspond, and the superscript 0 denoting the experimental values. The calculation of Γ_tr,*q*_ and Γ_rot,*q*_ is described in more detail in the following sections. It is worth noting that this optimization model is not limited to aqueous dispersions. Because the theoretical decay rates are calculated using experimental inputs for temperature (*T*), solvent viscosity (η), and refractive index (*n*), this model is immediately applicable to nanorods dispersed in other common solvents (e.g., ethanol, DMSO, etc.) without modification. Guided by work from Jayaraman and coworkers, who first introduced a genetic algorithm approach to interpret small-angle scattering measurements (“CREASE”),^36–39^ we defined the fitness function of individual *i* as

9 $$f_{i}\left(SSE_{i}\right)=X\left(SSE_{\max}-SSE_{i}\right)+Y$$

where SSE_max_ = max­(SSE_*i*_) is the maximum value of the SSE within the population of candidate solutions in a given generation. The coefficients *X* and *Y* are evaluated on the basis of the entire generation according to^37,40^

10 $$X=\left(C_{s}-1\right)\left[\frac{\max\left(SSE_{\max}-SSE_{i}\right)}{\max\left(SSE_{\max}-SSE_{i}\right)-\mathrm{avg}\left(SSE_{\max}-SSE_{i}\right)}\right]$$

and

11 $$Y=\left(1-X\right)\times \mathrm{avg}\left(SSE_{\max}-SSE_{i}\right)$$

where max(···) and avg(···) denote the maximum and average values of the argument across the entire population at a given generation, respectively (i.e., evaluated for all values of *i*). Following Beltran et al.,^37^ we set *C*_*s*_ = 10.

The fitness of the initial population is evaluated according to eq 9, and the probabilities *p*_*C*_ and *p*_*M*_ are initialized to be 0.6 and 0.001, respectively.^37^ *p*_*C*_ and *p*_*M*_ are adjusted periodically during the GA optimization to ensure that sufficient genetic diversity exists within the population of potential solutions, where the reciprocal genetic diversity is defined as

12 $$G_{d}^{-1}=\frac{1}{\max\left(f_{i}\right)\times N_{p}}\overset{N_{p}}{\underset{i=1}{\sum }}f_{i}$$

Similar to the approach taken by Beltran et al.,^37^ we increase *p*_*M*_ and decrease *p*_*C*_, respectively, by a factor of 1.1 if $G_{d}^{-1}>0.85$ . Similarly, if $G_{d}^{-1}<0.005$ , we increase *p*_*C*_ and decrease *p*_*M*_ by a factor of 1.1. This evaluation is performed every 50 generations and ensures a sufficient number of individuals with intermediate values of fitness are present in the population at each generation. After the fitness is evaluated for a given generation, the fittest candidate is selected to progress to the subsequent generation. For all other individuals in the population, they are mutated or undergo crossover with probabilities *p*_*M*_ and *p*_*C*_. Individuals that do not undergo crossover or mutation are retained for the next generation with a probability proportional to their fitness. The process is repeated for a total of 1000 generations, with the fittest individual at that point selected as the optimal solution.

## Results

### UV–Visible Spectroscopy

The optical absorption peak resulting from the longitudinal surface plasmon resonance (LSPR) is commonly used to estimate the length (*L*) and diameter (*D*) of nanorods. Our selection of nanorod dimensions was deliberate to minimize the absorption cross-section near the laser wavelength used for light scattering measurements (λ ≈ 637 nm) and to avoid similar effects on the scattering efficiency of the nanoparticles. We confirmed that the absorption spectra for AuNRs 980, AuNRs 900, and AuNRs 750 are situated away from the laser wavelength, as shown in Figure 3. The transverse surface plasmon resonance (TSPR) and longitudinal surface plasmon resonance (LSPR) peaks for AuNRs 980 are observed at 510 and 980 nm, respectively, with minimum absorption occurring around the laser wavelength. For AuNRs 900, the TSPR peak is obtained at 510 nm, and the LSPR produces a relatively broad, nonuniform peak between 850 and 1000 nm. The shape of the LSPR peak may be indicative of the polydispersity in nanorod lengths. The TSPR peak for AuNRs 750 is obtained at 510 nm, while two distinct LSPR peaks are observed, one around 750 nm and the other around 970 nm. The appearance of two peaks suggests multiple populations of AuNRs, perhaps due to the aggregation of a fraction of the particles.

**Figure 3:**
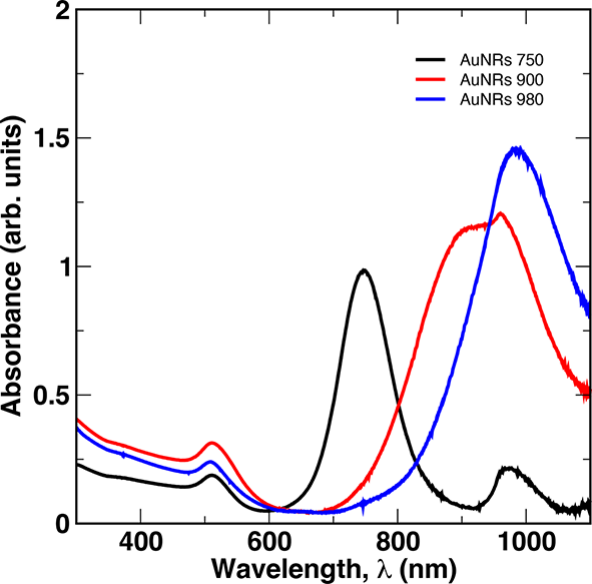
UV–vis characterization of the AuNRs samples. Curves correspond to AuNRs 750 (black), AuNRs 900 (red), and AuNRs 980 (blue). The peak near λ = 510 nm corresponds to the transverse surface plasmon resonance due primarily to the diameter of the rods, while the location of the LSPR increases from ca. 750 to 980 nm as the particle length increases.

### Electron Microscopy

We next conducted a comprehensive analysis of the three dimensions of AuNRs using a combination of Transmission Electron Microscopy (TEM) and Scanning Electron Microscopy (SEM) to measure the actual particle size distribution and compare it to our DDLS measurements. Figure 4 contains representative TEM micrographs for the three AuNR samples. Representative SEM micrographs are provided in the Supporting Information. A minimum of 100 rods was used to evaluate the nanorod size distributions. As is typical for standard AuNR synthetic procedures, a small amount of spherical impurities can be observed in the TEM images of AuNRs 980 and 750 (Figure 4a and c, respectively). These impurities produce a shoulder in the TSPR peak near λ ≈ 500 nm. In addition, AuNRs 900 show two distinct populations of nanorods: short, slender nanorods (6 × 39 nm) alongside a population of relatively wide, longer rods (15 × 68 nm). These two populations were analyzed separately in the Supporting Information. Finally, although AuNRs 750 showed two peaks in UV–vis at longer wavelengths, electron microscopy observed only nanorods and spherical impurities, implying that the peak observed near 970 nm may be due to a small number of AuNR aggregates in solution. The potential impacts of this are described later.

**Figure 4:**
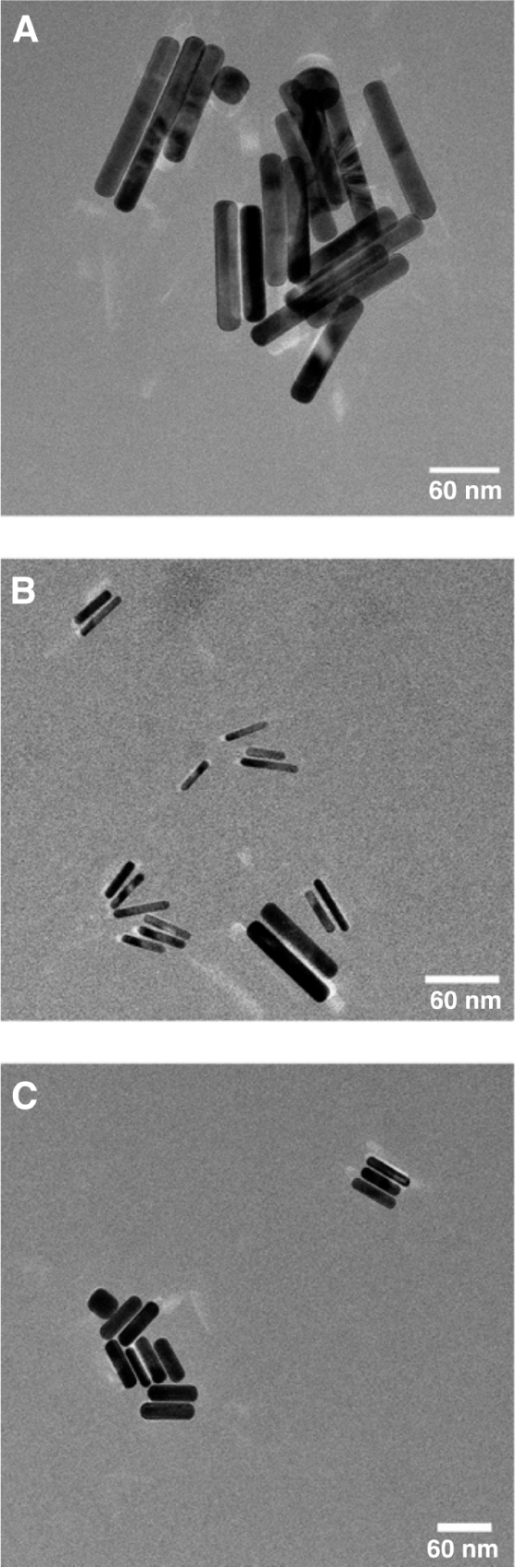
Representative TEM micrographs for (A) AuNRs 980, (B) AuNRs 900, and (C) AuNRs 750. All scale bars are 60 nm.

The mean length and diameter of the nanorods, along with their corresponding standard deviations, are shown in Table 1 for each microscopy technique. The corresponding size distributions can be found in Supporting Information. For comparison, the manufacturer specifications for the dimensions are also provided. The TEM and SEM results agree well with each other. However, Table 1 shows significant discrepancies between the manufacturer’s specified dimensions and our TEM/SEM measurements. In fact, for AuNRs 980, both SEM and TEM results yield dimensions of the nanorods that are about 70–80% larger than the manufacturer’s stated dimensions. For AuNRs 900, the reported lengths were approximately 40% larger than those reported by the manufacturer, while for AuNRs 750, the nanorod dimensions were approximately 50–60% larger than the specifications. At the same time, the values of the aspect ratio ν = *L*/*D* calculated using the dimensions obtained by electron microscopy were in very close agreement with the values of ν provided by the manufacturer. The quantitative agreement between the measured and manufacturer’s values of ν may be due to the fact that the LSPR of AuNRs depends very strongly on ν, and different values of *L* and *D* may produce similar LSPR wavelengths provided their aspect ratios are similar—suggesting that care should be taken when ascribing dimensions to AuNRs on the basis of their optical extinction alone. Nevertheless, it is possible to estimate the LSPR wavelength^32^ using ν as λ_*LSPR*_ ≈ 390.33 + 100.87ν. From the TEM dimensions from Table 1, this expression yields λ_*LSPR*_ = 975 nm (AuNRs 980), λ_*LSPR*_ = 844 nm (AuNRs 900, large nanorods), λ_*LSPR*_ = 1062 nm (AuNRs 900, short nanorods), and λ_*LSPR*_ = 763 nm (AuNRs 750). These estimates are in good agreement with the UV–vis spectra shown in Figure 3 and also suggest that the broad LSPR peak observed for AuNRs 900 is, indeed, due to the two populations of nanorods, with the short, slender rods giving rise to absorbances near 1000 nm. They also suggest that the small peak observed for AuNRs 750 near 980–1000 nm could be due to either a small fraction of high aspect ratio nanorods, small aggregates of AuNRs, or both.

**Table 1:** Summary of AuNR Dimensions and Aspect Ratio (*ν* = *L*/*D*) from TEM/SEM Analysis and as Provided by the Manufacturer

	**TEM**			**SEM**			**Manufacturer**		
Sample	*L* (nm)	*D* (nm)	ν	*L* (nm)	*D* (nm)	ν	*L* (nm)	*D* (nm)	ν
AuNRs 980	104 ± 15	18 ± 1	5.8	100 ± 16	17 ± 2	5.9	59	10	5.9
AuNRs 900^a^	68 ± 7	15 ± 1	4.5	69 ± 7	14 ± 2	4.9	50	10	5.0
AuNRs 750	55 ± 5	15 ± 3	3.7	52 ± 5	15 ± 2	3.5	35	10	3.5

a TEM analysis of AuNRs 900 revealed a bimodal size distribution with populations at 15 × 68 nm (dominant, reported here) and 6 × 39 nm (minor). The complete size distribution histograms are provided in the Supporting Information.

### Depolarized Dynamic Light Scattering (DDLS)

In contrast to EM, DDLS samples a large number of particles. Based on the concentration of our AuNRs and estimated scattering volumes at 90°, we estimate that our measurements probe on the order of 10^6^ AuNRs. Figure 5a shows normalized VV autocorrelation functions for AuNRs 980. From left to right, as indicated by the arrow, the value of *q* decreases, and the DDLS measurements probe increasingly larger length scales. VV autocorrelation functions measured by DDLS were found to be bimodal for all three samples of AuNRs, especially at small *q*. Indeed, as *q* decreases, *g* ^(2)^_VV_(τ)* transitions from exhibiting a single decay to showing two clear decays. The amplitude of the slow mode of the VV autocorrelation functions increased from 0.38 at small angles to 0.48 at large angles. Similar behavior of the VV autocorrelation functions was observed from AuNRs 900 and AuNRs 750, except the amplitude of the slow mode of AuNRs 750 decreased with increase of the angle and was much higher between 30° and 65° as will be discussed later. The autocorrelation functions for AuNRs 900 and AuNRs 750 and graphs of the mode amplitudes for all AuNRs are provided in Supporting Information.

**Figure 5:**
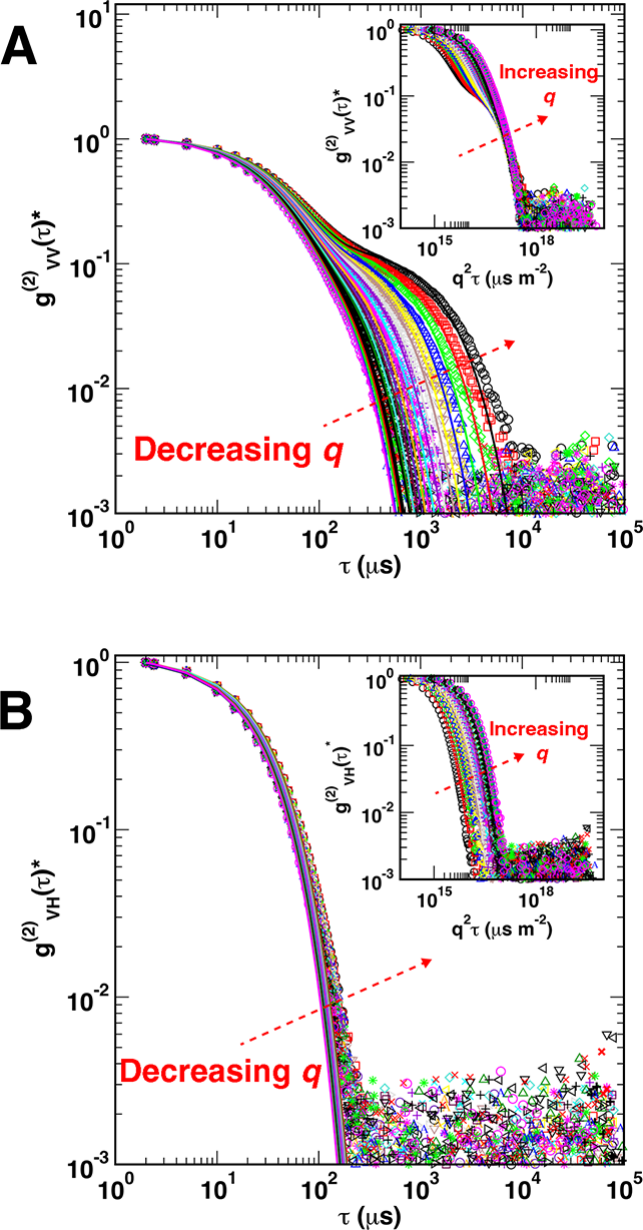
Representative DDLS measurements of AuNRs 980 as a function of scattering vector *q* from *q* ≈ 0.0068 nm^–1^ to *q* ≈ 0.025 nm^–1^. The graphs show (A) the normalized VV autocorrelation function, *g* ^(2)^_VV_(τ)*, coherence parameter *f* = 0.55–0.78, and (B) the normalized VH autocorrelation function, *g* ^(2)^_VH_(τ)*, *f* = 0.50–0.78. The arrows in the main panels indicate the direction of the variation in *q*. The insets of both figures show the scaling of *g* ^(2)^(τ)* with *q* ^2^τ, with both inset arrows in the direction of increasing *q*.

The bimodal nature of the autocorrelation functions is unexpected for these nanorods, as according to theory,^34,35^ the contributions due to rotational motion of short cylinders are not expected to be sufficiently significant to be seen in VV autocorrelation functions for cylinders of lengths *L* such that *qL* < 3–5. Indeed, for our longest nanorods, *L* ∼ 100 nm (AuNRs 980) and *qL* = 2.5. Additionally, the rotational diffusion contributions are expected to be more prevalent at larger angles, whereas we observe a more prominent fast VV mode at small angles.^27^ The presence of a significant fast VV mode may be due in part to a large depolarization ratio Δ = *I*_VH_/*I*_VV_, where *I*_VH_ and *I*_VV_ are the intensities of scattered light in the VH and VV measurements, respectively. AuNRs have depolarization ratios that vary as a function of wavelength due to plasmonic effects.^41,42^ As noted by Fytas et al.,^42^ the VV measurement contains an isotropic diffusion term which is the origin of the slow mode we observe in the VV measurements. However, the VH signal also contributes to the intensity of the VV measurement. If Δ is small, this contribution is negligible, and only a single decay is expected. As we show in SI (Section 7), for our nanorods Δ is large at λ = 637 nm, and, as a result, we observe a fast mode in the VV signal that may represent a mixture of translational and rotational motions. However, determining the specific nature of this decay of the autocorrelation is challenging and warrants future study. Nevertheless, the presence of the fast VV mode can be mitigated by taking measurements at a different wavelength where Δ is small (cf. Figure S16), but a low depolarization ratio will also result in a weak depolarized signal, which complicates DDLS analysis.

To gain insight into the physical processes responsible for various decays in the multimodal autocorrelation functions, we analyzed *g* ^(2)^_VV_(τ)* plotted against *q* ^2^τ. The inset of Figure 5a shows *g* ^(2)^_VV_(τ)* vs *q* ^2^τ for AuNRs 980 at all scattering angles. As indicated by the arrow, curves from left to right correspond to increasing values of *q* (i.e., higher angles). This scaling accentuates the difference between a *g* ^(2)^_VV_(τ)* describing purely translational motion and one describing something else, possibly some combination of translational and rotational motion. Indeed, for monodisperse spherical particles *g* ^(2)^_VV_(τ)* is unimodal due to an inability to resolve rotational motion—resulting in *q* ^2^ scaled curves collapsing onto a single master curve. In contrast, for longer nanorods, the autocorrelation function is expected to be bimodal and reflect both translational and rotational motion of the particles. Consequently, the collapse is only observable for the decay mode (and/or within the range of angles) where the decay is solely attributed to translational diffusion.

The inset of Figure 5a shows two distinct behaviors. The first decay *g* ^(2)^_VV_(τ)* of exhibits no scaling across the scattering angles. This suggests the fast VV mode might correspond to some contributions from both translational and rotational diffusion of the nanorods or other physical processes. In contrast, the second decay of *g* ^(2)^_VV_(τ)* exhibits a clear scaling for all angles, with all autocorrelation functions converging together. This observation suggests that the motion probed by the second mode of *g* ^(2)^_VV_(τ)* is primarily translational, and for this reason, the second mode was utilized to determine the translational diffusion coefficient *D*_tr_. Similar analysis was conducted on AuNRs 900 and AuNRs 750. The second mode of the scaled *g* ^(2)^_VV_(τ)* overlapped for all scattering angles for AuNRs 900; hence, the entire angle range was considered to determine *D*_tr_ for this sample as well (Figure S10a, Supporting Information). However, for AuNRs 750, the second mode of *g* ^(2)^_VV_(τ)* for the angle range 30° to 65° did not exhibit overlapping behavior. As a result, this lower angle data was excluded from the analysis when determining *D*_tr_ for AuNRs 750 (Figures S4a, S5, Supporting Information), which was also justified by a poor quality of the AuNRs 750 autocorrelation functions at these small angles.

The normalized VH autocorrelation functions for AuNRs 980 are depicted in Figure 5b and show a unimodal decay. Curves from left to right correspond to decreasing values of *q*. *g* ^(2)^_VH_(τ)* should reveal a constant contribution to the rotational diffusion coefficient across all scattering angles. Indeed, the normalized *g* ^(2)^_VH_(τ)* for the entire scattering angle range shows a consistent and largely overlapped pattern as depicted in the figure. Similar behavior was observed for AuNRs 900 and AuNRs 750 (Figures S10b and S5b, Supporting Information). In contrast to the behavior observed for the VV signal (cf., Figure 5a), when plotted against *q* ^2^τ, *g* ^(2)^_VH_(τ)* shows clearly separated functions moved to the right as the scattering angle increases indicating the presence of rotational diffusion contributions as anticipated (inset of Figure 5b).

### DDLS Data Analysis

To determine translational and rotational diffusion coefficients, we analyzed the angular dependence of the relaxation decay rates obtained from the VV and VH autocorrelation functions. Figure 6 plots relaxation rates of both decays of the VV autocorrelation functions as a function of *q* ^2^ for (a) AuNRs 980, (b) AuNRs 900, and (c) AuNRs 750, but only for the values of *q* that showed pure translational motion (i.e., for which the autocorrelation functions superimposed when plotted versus *q* ^2^τ). The entire *q*-dependence for AuNRs 750 can be seen in Figure S6 in the Supporting Information.

**Figure 6:**
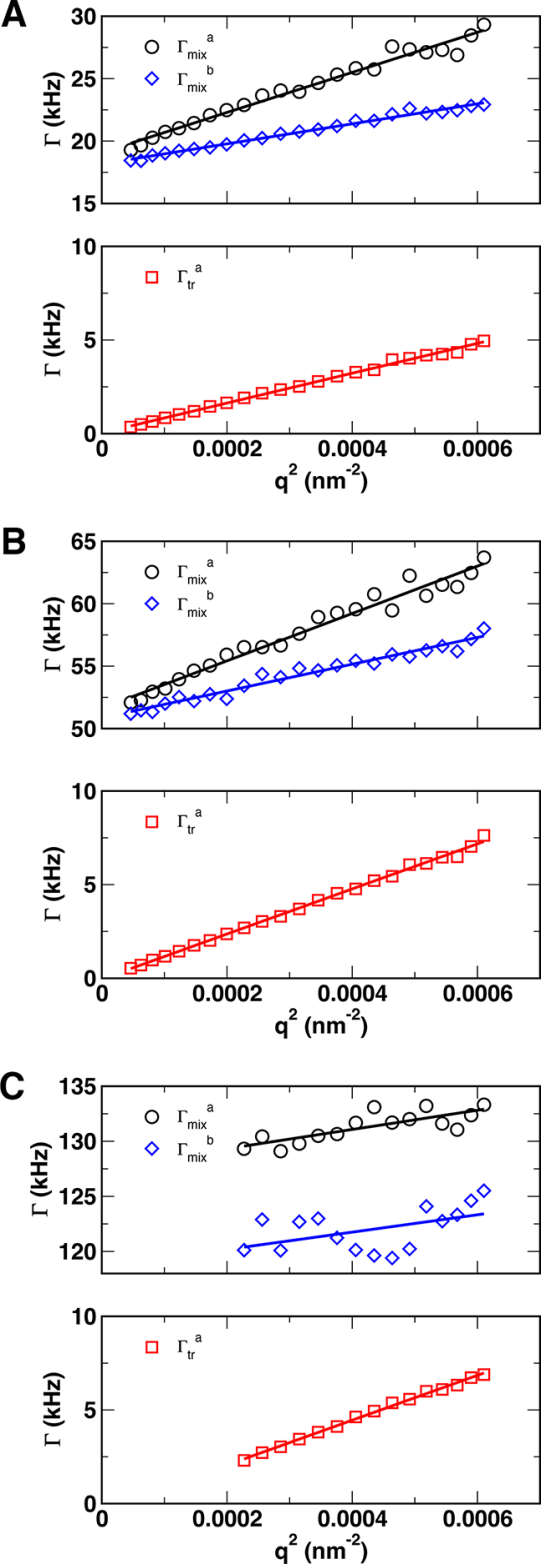
Relaxation rates Γ as the function of *q* ^2^ for (A) AuNRs 980, (B) AuNRs 900, and (C) AuNRs 750. In each panel, the top graph plots the mixed relaxation rate Γ_mix_ = Γ_tr_ + Γ_rot_, while the bottom graph plots only the translational relaxation rate, Γ_tr_^a^. Γ_mix_^a^ and Γ_mix_^b^ refer to mixed relaxation rates taken from the fast VV mode and VH, respectively. Solid lines are least-squares fits to the data.

Black and blue points correspond to Γ_mix_ taken from the fast VV mode and VH signals (Γ_mix_^a^ and Γ_mix_^b^respectively), while the red points show Γ_tr_ obtained from the VV signal $\left(\Gamma_{tr}^{a}\right)$ . Since the physical nature of the main decay of the VV data is well understood as translational motion, and its autocorrelation functions overlapped when scaled by *q* ^2^ (cf., Figure 5), we first focused on the *q*-dependence of the main decay of the VV signal $\left(\Gamma_{tr}^{a}\right)$ and *q*-dependence of the only decay of the VH signal to obtain the translational and rotational diffusion coefficients of the nanorods. *D*_tr_ was obtained from the average slope of $\Gamma_{tr}^{a}\left(q^{2}\right)$ and Γ_mix_^b^(*q* ^2^), while *D*_rot_ was obtained from the intercept of Γ_mix_^b^(*q* ^2^), since Γ_mix_=Γ_tr_ + Γ_rot_ = *D*_tr_ *q* ^2^ + 6*D*_rot_. The fast mode of the VV signal was initially neglected and will be discussed in more detail later. Additionally, the estimated *D*_tr_ from the higher angle *q*-dependence of AuNRs 750 data is expected to be somewhat off as $\Gamma_{tr}^{a}\left(q^{2}\right)$ had a nonzero intercept, potentially due to the plasmonic effects discussed above.

The translational and rotational diffusion coefficients are related to the length (*L*) and aspect ratio (ν) of the AuNRs according to

13 $$D_{tr}=\left(\frac{k_{B}T}{3\pi \eta L}\right)F\left(\nu \right)$$

14 $$D_{\mathrm{rot}}=\left(\frac{3k_{B}T}{\pi \eta L^{3}}\right)G\left(\nu \right)$$

where *k*_*B*_ is Boltzmann’s constant, *T* is the solution temperature, η is its viscosity, and *F*(ν) and *G*(ν) are model-dependent shape functions of the aspect ratio. Three distinct geometrical models were used in this study: the straight cylinder model (SC), the prolate ellipsoidal model (PE), and the spherocylinder model (SP). Predictions for straight cylinders are made by extrapolating polynomials derived by de la Torre et al. from simulation results. These extrapolations remain applicable for aspect ratios ranging from 2 to 20 ^43,44^ and, more recently, have been extended for aspect ratios as low as 0.1.^45^ While we utilize the polynomial extrapolations by de la Torre et al. for the SC model, we note that recent boundary element methods, particularly the work of Aragon and Flamik,^46^ provide high-precision solutions for cylinders with flat, open, and hemispherical ends that can be utilized for DDLS analysis. Additionally, software packages such as ZENO^47^ enable hydrodynamic modeling of complex shapes. These modern computational frameworks offer more refined predictions that are particularly valuable for characterizing particles with higher aspect ratios or more complex geometries. For the moderate aspect ratios investigated here (ν ≈ 3–6), the employed relations provide sufficient accuracy. Extrapolations from de la Torre et al. result in the following shape functions for a straight cylinder:

15 $$F_{SC}\left(\nu \right)=\ln\left(\nu \right)+0.312+\frac{0.565}{\nu }-\frac{0.1}{\nu^{2}}$$

16 $$G_{SC}\left(\nu \right)=\ln\left(\nu \right)-0.662+\frac{0.917}{\nu }-\frac{0.05}{\nu^{2}}$$

Similarly, expressions for prolate ellipsoids can be obtained from the work of Perrin:^28^

17 $$F_{PE}=\nu S\left(\nu \right)$$

18 $$G_{PE}\left(\nu \right)=\frac{\nu \left[\left(2\nu^{2}-1\right)S\left(\nu \right)-\nu \right]}{2\left(\nu^{2}-1\right)}$$

where

19 $$S\left(\nu \right)=\frac{\ln\left[\nu +\sqrt{\nu^{2}-1}\right]}{\sqrt{\nu^{2}-1}}$$

Finally, the shape functions for spherocylinders can be described by^48^

20 $$F_{SP}\left(\nu \right)=\ln\left(\nu \right)+\overset{5}{\underset{i=0}{\sum }}a_{i}\nu^{-i}$$

where *a*_0_ = 0.3863, *a*_1_ = 0.6863, *a*_2_ = −0.06250, *a*_3_ = −0.01042, *a*_4_ = −0.000651, and *a*_5_ = 0.0005859, and:

21 $$G_{SP}\left(\nu \right)=\ln\left(\nu \right)+2\;\ln\left(2\right)-\frac{11}{6}+\frac{\ln\left(2\right)}{\ln\left(1+\nu \right)}\left(\frac{1}{3}-2\;\ln\left(2\right)+\frac{11}{6}-\overset{6}{\underset{i=1}{\sum }}a_{i}\right)+\overset{6}{\underset{i=1}{\sum }}a_{i}\nu^{-i/4}$$

where *a*_1_ = 13.04468, *a*_2_ = −62.6084, *a*_3_ = 174.0921, *a*_4_ = −218.8365, *a*_5_ = 140.26992, and *a*_6_ = −33.27076.

A large challenge in analyzing DDLS measurements to determine AuNRs dimensions is that *a priori* knowledge of certain aspects of the AuNRs dimensions and certain assumptions are necessary to extract both *D* and *L*. As noted in Table 1, this information may not always be available or accurate and may change if ligands or polymers are attached to the surface of the nanoparticles. To overcome this challenge, we analyzed our DDLS measurements using three approaches and the expressions in eqs 13–21.

#### Approach 1: Solving for Aspect Ratio

In the first approach, we combined eqs 13 and 14, while substituting the appropriate model-dependent forms of *F*(ν) and *H*(ν) = *G*(ν)/*F*(ν) (eqs 15–21). This reduced the analysis to a single unknown, the aspect ratio ν, which was found by numerically solving the combined expression for ν. Finally, the length *L* was found from eq 13, and the diameter calculated as *D* = *L*/ν. A summary of the diffusion coefficients and AuNRs dimensions obtained from this approach is presented in Table 2.

**Table 2:** Translational (*D*_tr_) and Rotational (*D*_rot_) Diffusion Coefficients, and the Length (*L*) and Diameter (*D*) of AuNRs Samples Obtained Using Approach 1 for the Straight Cylinder (SC), Prolate Ellipsoid (PE), and Spherocylinder (SP) Models

			SC		PE		SP	
Sample	*D*_*tr*_ (cm^2^/s)	*D*_rot_(kHz)	*L* (nm)	*D* (nm)	*L* (nm)	*D* (nm)	*L* (nm)	*D* (nm)
AuNRs 980	7.99 × 10^–8^	3.03	110	29	137	30	122	29
AuNRs 900	1.14 × 10^–7^	8.48	79	20	98	21	87	20
AuNRs 750	9.92 × 10^–8^	19.77	35	39	56	46	42	60

Following this approach, for AuNRs 980 and AuNRs 900, the straight cylinder (SC) model seemingly produces results that are most consistent with the TEM and SEM findings, while the PE model shows the most disagreement. However, for all three models, there is significant deviation between the values from DDLS and those obtained by electron microscopy. For example, for AuNRs 980, DDLS overestimates the length of the AuNRs by approximately 6 nm in the SC model, 18 nm in the SP model, and 33 nm in the PE model (compared to TEM). Similarly, the diameters of the AuNRs are approximately 11–12 nm larger than the values obtained from electron microscopy. Similar trends persist for the AuNRs 900 sample. To some extent, the disagreement between values of *D* from DDLS and electron microscopy can be partially attributed to the presence of a 3.6 nm surfactant bilayer, not seen by EM, that surrounds the nanoparticles,^14^ adding approximately 7.2 nm to *D* and *L*. The disagreement between DDLS and electron microscopy may also be due, in part, to solution effects (e.g., hydrodynamic interactions, bound water, etc.) that are not present in microscopy samples. In addition, since light scattering probes ensemble average of all particles in the scattering volume while microscopy analyzes only a specific frame of a subset of these particles, some differences in the deduced size distributions for a polydisperse sample are expected. For AuNRs 750, the values obtained by DDLS differ significantly from electron microscopy and imply that the nanoparticles are more isotropic in shape (i.e., ν ≈ 0.73–1.2). This result is likely due to the noisy *q* ^2^-dependence of Γ_mix_^b^ seen in Figure 6c, which contributed to extremely high value of *D*_rot_. Previous reports in the literature suggest that *D*_rot_ is especially sensitive to nanorod length.^8^

#### Approach 2: Aspect Ratio Guess

Our second approach to estimating the nanoparticle dimensions, called the aspect ratio guess method, followed the work of Glidden and Muschol.^8^ However, in contrast to that work, we performed DDLS measurements at multiple angles and used the *q* ^2^-dependence of relaxation rates to obtain *D*_tr_ and *D*_rot_, as discussed above. This approach provided the length of the AuNRs assuming a single, monodisperse population of nanoparticles. Glidden and Muschol^8^ calculated the ratio *H*(ν), noting that its value was relatively constant as a function of ν for ν ∈ [2, 20]. On the basis of eqs 13 and 14, the length was obtained as

22 $$L=q^{-1}\sqrt{54\left(\frac{\Gamma_{tr}}{\Gamma_{\mathrm{rot}}}\right)H\left(\nu \right)}$$

We modified the approach by using *q* ^2^-dependence of Γ_tr_ to rewrite eq 22 as

23 $$L=3\sqrt{\left(\frac{D_{tr}}{D_{\mathrm{rot}}}\right)H\left(\nu \right)}$$

We then used an initial guess for ν, based on the dimensions provided by the manufacturer, to evaluate *H*(ν) in eq 23 and, therefore, the corresponding length *L*. For AuNRs with a manufacturer-specified aspect ratio ν, we would use the range of aspect ratios encompassing ν to account for the effects of polydispersity within the sample. The length from eq 23 was, in turn, used to calculate the value of *F*(ν):

24 $$F\left(\nu \right)=\left(\frac{3\pi \eta L}{k_{B}T}\right)D_{tr}$$

This value of *F*(ν) was plotted along with theoretical *F*(ν) values (from eq 15 or 17 or 20) for a range of ν, which encompassed the range of our initial guess. The point of intersection between theoretical *F*(ν) and actual *F*(ν), which we call ρ, indicates how close we are to the guessed value. For instance, if we were to start with the guess ν = 3 and the point of intersection ρ = 3.2, this would imply that our guess was close, and we would iterate the initial guess until the guess would be equal to the point of intersection.

The results of this second approach were found to be very similar to the results of Approach 1 (Table 2) for AuNRs 980 and AuNRs 900 using the SC, PE, and SP models, and for AuNRs 750 using just the SC and PE models. Approach 2 did not converge for the AuNRs 750 sample when using the SP model. All results from Approach 2 appear in the Supporting Information (Table S1).

Both Approach 1 and Approach 2 are based on the assumption that the AuNRs are monodisperse. While standard procedures for AuNRs synthesis result in particles with highly uniform diameters, some degree of polydispersity exists in the length and, therefore, in the aspect ratio. The dispersity of AuNRs samples is also affected by a small amount of spherical impurities that frequently form as the nanorods grow, leading to two populations of particles—each with their own translational and rotational relaxation rates.

#### Approach 3: Genetic Optimization

Finally, we applied a genetic optimization algorithm (GA) to interpret our DDLS measurements and evaluated its effectiveness as compared to the two methods described above. A motivation for using the GA was that it would not require any *a priori* knowledge of the AuNRs’ dimensions or assumptions regarding the value of *H*(ν) and could further determine which model (SC, PE, or SP) best describes the experimentally observed relaxation rates. By optimizing the choice of *L* and *D*, this approach is also amenable to samples with some small degree of polydispersity in their dimensions since it acts to minimize the difference between experimental and theoretical values rather than directly matching them. Note that the data set used for the GA approach differs from that used in the two approaches described above. Whereas the first two approaches rely on the measured *q*-dependence of the relaxation rates to obtain diffusion coefficients, the GA approach instead acts to optimize choices of *L* and *D* that best match the individual relaxation rates at each value of *q*. Table 3 presents the results of applying the GA to a data set composed of $\Gamma_{tr}^{a}\left(q\right)$ and Γ_rot_^b^(*q* ^2^) = Γ_mix_^b^(*q* ^2^) - Γ_tr_^a^(*q* ^2^) (i.e., excluding the fast mode in the VV measurement).

**Table 3:** Length (*L*) and Diameter (*D*) of AuNRs Samples Obtained Using a Genetic Optimization Algorithm (GA) for the Straight Cylinder (SC), Prolate Ellipsoid (PE), and Spherocylinder (SC) Models

	SC		PE		SP	
Sample	*L* (nm)	*D* (nm)	*L* (nm)	*D* (nm)	*L* (nm)	D (nm)
AuNRs 980	114	26	140	28	129	24
AuNRs 900	82	18	101	18	89	18
AuNRs 750	40	30	64	30	54	30

The GA optimization was applied separately for each of the three geometrical models, and its effectiveness was assessed on the basis of the agreement with the measured relaxation rates across all values of *q*. For AuNRs 980, the best candidates (cf. Table 3) resulted in average differences between the experimental and theoretical relaxation rates of (ΔΓ_tr_, ΔΓ_rot_) = (2.6%,0.9%) for the SC model. Similarly, for the best PE and SP candidates, average differences of (2.5%, 0.8%) and (3.4%, 4.0%) were observed, respectively.

Although the PE model predicted relaxation rates that were closest to those measured, the prolate ellipsoid is a poor choice of a geometrical model for nanorods (e.g., based on known shapes from electron microscopy). Because of this, the dimensions produced by the SC model were much closer to the values known from electron microscopy—suggesting a straight cylinder may be the best model for long, thin Au nanorods. Applying the GA approach to AuNRs 900 produced similarly good agreement, with average differences between the experimental and theoretical values in the SC, PE, and SP models of (ΔΓ_tr_, ΔΓ_rot_) = (2.2%, 0.7%), (1.7%, 0.7%), and (1.9%, 0.9%), respectively. As with AuNRs 980, we find that the PE model is a poor choice geometrically, and the SC model produces the best agreement with dimensions obtained by electron microscopy. Finally, although AuNRs 900 was found to contain two populations of AuNRs, the GA algorithm produced dimensions that were most similar to the dominant population of longer nanorods. This may be due, in part, to the fact that larger nanorods are expected to scatter light more strongly and, as a result, may contribute more to the DDLS autocorrelation functions.

As noted above, both Approaches 1 and 2 had difficulty producing acceptable dimensions for sample AuNRs 750 and typically predicted more symmetric particles with ν ≈ 0.73–1.2 as a result of overpredicting the diameter of the particles. In fact, the SP model did not converge at all. At the same time, whereas Approach 2 did not converge for AuNRs 750, the GA did not experience any difficulty estimating the dimensions of this sample. The GA approach was able to find optimal dimensions that were more rod-like in shape (ν ≈ 1.3 to 2.1) for this sample, although similar to the other analysis methods, it consistently overpredicted the diameter by a factor of about 2. In contrast to samples AuNRs 980 and AuNRs 900, the GA approach found that the spherocylinder model more accurately described the dimensions of the AuNRs 750 than did either the SC or PE models. Given the relatively short dimensions of these particles as compared to the other two samples, it may be that as the nanorod length becomes shorter, the end caps of the nanorods contribute more to the translational and rotational dynamics of the particles, making the spherocylinder a more accurate model than either the prolate ellipsoid or straight cylinder.

## Discussion

### Excluding the Fast VV Mode

A summary of our results using all three approaches, and all three geometric models, is shown in Figure 7 for (a) AuNRs 980, (b) AuNRs 900, and (c) AuNRs 750. These results were obtained with the fast VV mode excluded. For each nanorod sample, the average values of length and diameter are overlaid as gray (TEM) and blue (SEM) boxes, with the range of the box denoting one standard deviation, and the dashed line representing the average value. Comparisons between TEM and the best candidate from the GA optimization are illustrated, to scale, to the right of each graph. In Figure 7, we observe that for AuNRs 980 and AuNRs 900, the three approaches produce dimensions that are relatively close to one another, with GA slightly overestimating the length of the particles in comparison to the other two approaches. All three approaches also over predict the diameter of the nanorods (with GA performing slightly better than the other two approaches), which may be due to the presence of a thin layer (≈ 3.6 nm) of CTAB on the surface. We also find that the SC model produces results through all three approaches that are most consistent with electron microscopy. In contrast, although all three approaches produce similar values for the longer dimension of AuNRs 750, the GA optimization consistently performs best, resulting in values of the shorter dimension of the particles that are closer to the value obtained from microscopy, although still a factor of 2 larger. Correspondingly, the graphical depictions of the GA prediction of AuNRs 750 dimensions show greater disagreement.

**Figure 7:**
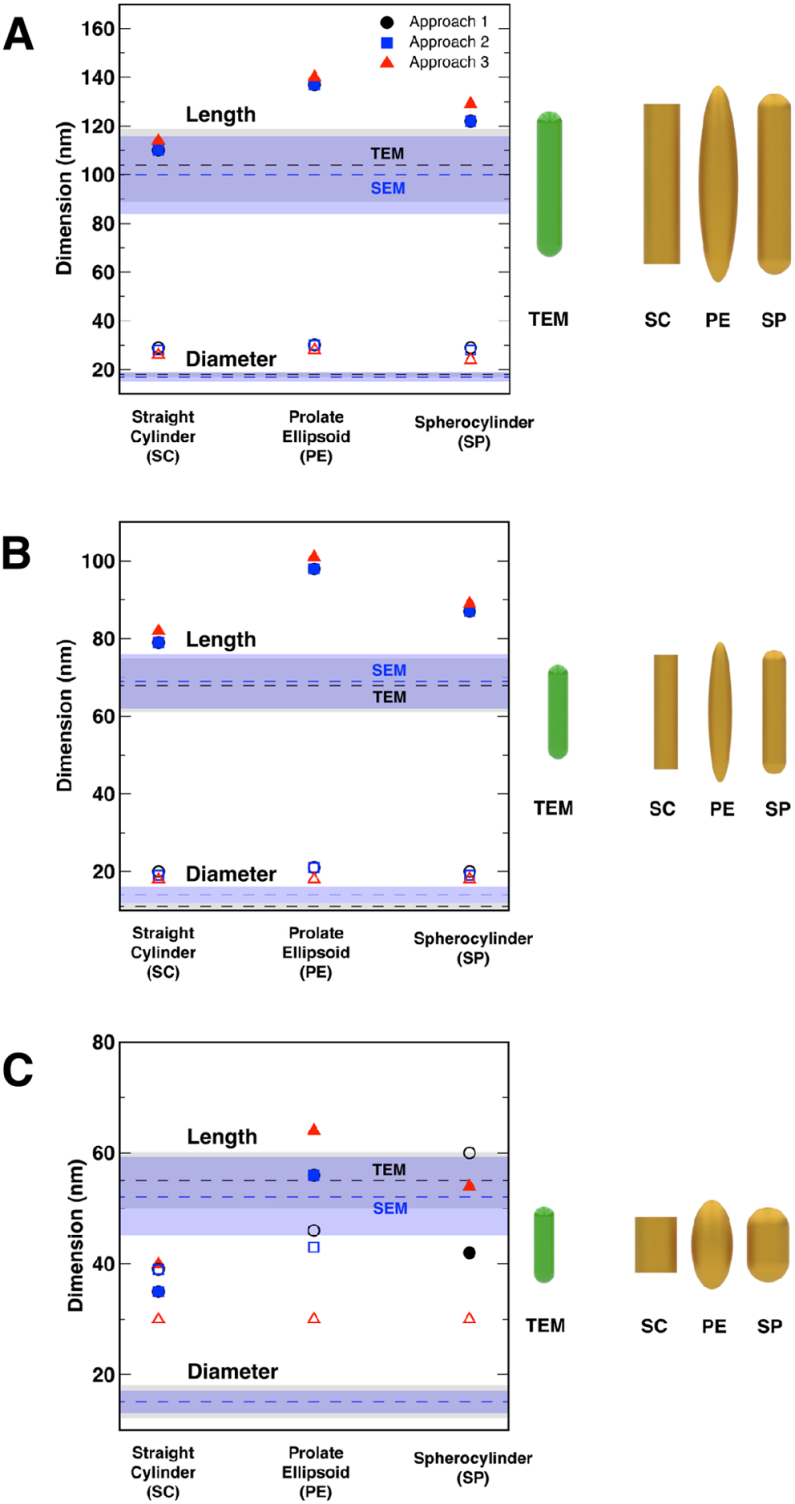
Comparison of AuNRs dimensions obtained by three different methods: Approach 1 (solving for aspect ratio, circles), Approach 2 (aspect ratio guess, squares), and Approach 3 (genetic optimization, triangles) for (a) AuNRs 980, (b) AuNRs 900, and (c) AuNRs 750. The gray and blue bands depict one standard deviation from the average dimensions (dashed lines) obtained by TEM and SEM, respectively. Open and filled symbols correspond to values of diameter and length, respectively. The illustrations to the right of each panel are to scale and depict the comparison between a spherocylinder with dimensions obtained from TEM analysis, compared to the SC, PE, and SP results from Approach 3.

### Including the Fast VV Mode

In our analysis above, we considered only the variation of $\Gamma_{tr}^{a}$ (slow VV mode) and $\Gamma_{\mathrm{mix}}^{b}$ (VH decay) with *q* ^2^ using Approach 1 and Approach 2. *D*_tr_ was obtained as the average of the two slopes of those variations, and *D*_rot_ was obtained from the y-intercept of $\Gamma_{\mathrm{mix}}^{b}$ . The GA optimization directly utilized only $\Gamma_{\mathrm{tr}}^{a}$ and $\Gamma_{\mathrm{mix}}^{b}$ . As a result, we excluded $\Gamma_{\mathrm{mix}}^{a}$ , which describes the fast decay of the VV signal (i.e., the decay that did not scale with *q* ^2^τ). Despite the uncertain nature of the observed fast VV mode, we repeated the analysis described above by including the fast mode. In this case, we treated the system as an ensemble of nanorods where $g_{VV}^{\left(2\right)}{\left(\tau \right)}^{*}$ is bimodal and depends both on translational and rotational motions as indicated by eq 3. *D*_tr_ was obtained from the average of the slopes of all three relaxation rates ( $\Gamma_{tr}^{a}$ , $\Gamma_{\mathrm{mix}}^{a}$ and $\Gamma_{\mathrm{mix}}^{b}$ ) when plotted against *q* ^2^, while *D*_rot_ was obtained as the average of the two y-intercepts ( $\Gamma_{\mathrm{mix}}^{a}$ and $\Gamma_{\mathrm{mix}}^{b}$ ). The diffusion coefficients we obtained with the fast mode included are summarized in Table S2 for all three samples.

A summary of the dimensions for all three AuNRs samples using Approach 1 and 2, with the fast VV mode included, is contained in Tables S2 and S3, respectively. For all samples, the dimensions obtained are substantially less agreeable with TEM/SEM findings, as the fast VV mode has a different slope between the VH signal and main decay of the VV signal, as shown in Figure 6a and b. Fytas et al.^42^ also observed that DDLS can produce different diffusion coefficients depending on the measurement conditions and laser wavelength due to plasmonic effects. For AuNRs 980 and 900, the three analysis methods produce much smaller values of the diameter of the nanorods (*D* ≈ 9 nm) and slightly larger lengths, leading to an overprediction of ν. In contrast, the dimensions obtained for AuNRs 750 are similar to those obtained when the fast mode is excluded but still suffer from a significant overestimation of the diameter of the particles and aspect ratios ν ≤ 1. Although *D*_tr_ is similar for AuNRs 750 when the fast mode is included, Γ_rot_ is approximately 1 kHz faster. AuNRs 750 also suffers from relatively weak scattering intensities and a lower coherence factor. The slow mode decay rates that we measured may be influenced by a combination of weak scattering and plasmonic effects. We observe that the extrapolation of the AuNRs 750 relaxation rates,(*q* ^2^), to *q* = 0 (Figures 6c and S6) does not intercept the origin, which may be an indication of uncertainty in *D*_tr_. These factors combined may explain the different results produced by the two approaches for this sample.

Finally, we repeated a similar analysis using the GA algorithm by directly using Γ_tr_ and computing Γ_rot_ from the average of the mixed relaxation rates, $\Gamma_{\mathrm{rot}}\left(q\right)=\left[\Gamma_{\mathrm{mix}}^{a}\left(q\right)+\Gamma_{\mathrm{mix}}^{b}\left(q\right)\right]/2-\Gamma_{tr}^{a}\left(q\right)$ . When the fast mode from the VV signal was included, we observed a larger difference between experimental and theoretical relaxation rates by approximately 2–5%. Overall, we observed that inclusion of the fast mode resulted in slightly different predictions of the AuNRs’ diameters, but these predictions varied by no more than approximately 2–6 nm from those obtained by excluding the fast mode. Predictions of AuNRs’ lengths were generally smaller when the fast VV mode was included and differed by at most 12 nm from the predictions without the fast mode included. Notably, these differences were much smaller in Approach 3 than in Approaches 1 and 2, demonstrating, in part, the robustness of the genetic algorithm approach to interpreting DDLS measurements. A summary of GA results with the inclusion of the fast mode for all samples appears in Table S4 in the Supporting Information.

## Conclusions

In conclusion, we performed depolarized dynamic light scattering (DDLS) measurements on three separate Au nanorod samples, each with different optical characteristics and dimensions. Although the aspect ratio of the particles, calculated by electron microscopy, was in good agreement with the values provided by the nanorod manufacturer, we found significant disagreement between our measured dimensions and those provided by the manufacturer—suggesting their dimensions were estimated using the wavelength of the longitudinal surface plasmon resonance alone.

Traditionally, measuring Au nanorod dimensions by DDLS has required *a priori* knowledge of either the aspect ratio or the length of the rods, highlighting the difficulties that can be encountered if those dimensions are not available or unreliable. For that reason, we focused on multiangle DDLS measurements and their analysis using three techniques – Approach 1: Solving for Aspect Ratio, Approach 2: Aspect Ratio Guess, and Approach 3: Genetic Optimization – all with the goal of obtaining Au nanorod dimensions without any *a priori* knowledge of the dimensions.

Our results demonstrated that reasonable estimates of Au nanorod dimensions can be obtained using the three analysis approaches. In Approach 1, these estimates are obtained without any prior information by utilizing the *q*-dependence of the measured relaxation rates. Similarly, Approach 2 also utilizes the *q*-dependence of the relaxation rates while also incorporating the shape function *H*(ν) to refine the aspect ratio estimation. In contrast to Approaches 1 and 2, Approach 3 treats the *q*-dependence differently by directly utilizing the relaxation rates and optimizing AuNR dimensions to simultaneously match the relaxation rates across all angles. Although the three methods were in general agreement with one another, the genetic algorithm was able to obtain slightly better estimates for AuNRs 750, for which the light scattering data was noisy and difficult to analyze. These successes can enable robust characterization of nanorod dimensions without the need for electron microscopy or small-angle scattering, which can be prohibitively expensive or otherwise unavailable in certain research settings. They also emphasize the robustness of the DDLS technique in probing rotational and translational diffusion, and consequently deducing the apparent dimensions of less-than-perfect particles in solution.

Looking into the future, the genetic algorithm approach to analyzing DDLS data may be especially useful in characterizing polymer-grafted nanorods, since grafting polymers to the surface will slow translational and rotational motion of the particles and alter the aspect ratio of the nanoparticles in a way that is difficult, if not impossible, to observe by electron microscopy. Additionally, small-angle scattering measurements of polymer-grafted nanoparticles can be extremely difficult to interpret analytically due to the lack of appropriate form factors. Our GA method may be extended to analyze DDLS measurements of other shapes of nanoparticles, including when they are grafted with polymers. This extension relies only on the availability of suitable models of the rotational and translational diffusion rates of other geometries, which can be obtained from appropriate computer simulations (e.g., dissipative particle dynamics, dynamic Monte Carlo, or Brownian dynamics). Future measurements of these sorts of grafted nanoparticles may, for instance, be valuable tests of theories of polymer brush conformation and may provide mechanistic insight into the behavior of polymer-grafted nanoparticles in advanced materials.

## Supplementary Material



## Notes

Scanning electron micrographs, nanorod size distributions, detailed depolarized DLS measurement results, and depolarization ratio calculations (PDF)
