# Supplementary material for: Parameter-Free Determination of Au Nanorod Dimensions Using Depolarized DLS and Genetic Optimization
Source: J Phys Chem B. 2026 Jan 29;130(6):1960–73. doi: 10.1021/acs.jpcb.5c06410 (PMC12908119; doi:10.1021/acs.jpcb.5c06410)
Supplement: Supplementary file 1 [file jp5c06410_si_001.pdf]

Supporting Information:
Parameter-Free Determination of Au Nanorod Dimensions Using Depolarized DLS and Genetic Optimization

Nehal Nupnar[1], Geofrey Nyabere[2], Claire M. B. Bolding[1], Kiril A. Streletzky[2,#], Michael J. A. Hore[1,†]

[1]Department of Macromolecular Science and Engineering, Case Western Reserve University, Cleveland, OH 44106, United States.
[2]Department of Physics, Cleveland State University, Cleveland, OH 44114, United States.
[†]hore@case.edu
[#]k.streletzky@csuohio.edu

Contents

1 AuNRs 750 Data Analysis **S2**
1.1 Transmission Electron Microscopy . . . S2
1.2 Scanning Electron Microscopy . . . S2
1.3 Depolarized DLS . . . S2

2 AuNRs 900 Data Analysis **S5**
2.1 Transmission Electron Microscopy . . . S5
2.2 Scanning Electron Microscopy . . . S5
2.3 Depolarized DLS . . . S5

3 AuNRs 980 Data Analysis **S8**
3.1 Transmission Electron Microscopy . . . S8
3.2 Scanning Electron Microscopy . . . S8

4 Depolarized DLS Analysis Neglecting Fast VV Mode **S10**
4.1 Approach 2: Aspect Ratio Guess . . . S10

5 Depolarized DLS Analysis Using Fast VV Mode **S10**
5.1 Approach 1: Solving for Aspect Ratio . . . S10
5.2 Approach 2: Aspect Ratio Guess . . . S10
5.3 Approach 3: Genetic Optimization . . . S11

6 DDLS Mode Amplitudes **S11**

7 Calculation of the Depolarization Ratio (Δ) **S11**
7.1 Discrete Dipole Approximation . . . S11
7.2 Calculation of VV and VH Scattering Intensity . . . S12

1 AuNRs 750 Data Analysis

1.1 Transmission Electron Microscopy

Transmission electron microscopy (TEM) analysis was conducted on AuNRs 750 to characterize their size distribution and dispersity. The analysis revealed a homogeneous population of nanorods within the sample space. The size of these nanorods was found to be 15×55 nm as shown in Fig. S1. Representative TEM images are provided in the main text.

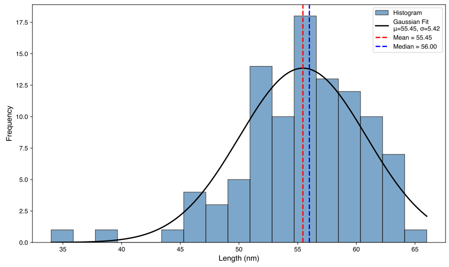

(a)

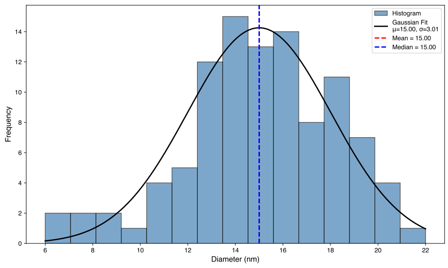

(b)

Figure S1: Histograms of Au nanorod (a) length and (b) diameter for sample AuNRs 750 obtained from TEM imaging. The black lines are fits to a Gaussian distribution, yielding average dimensions of $L = 55 \pm 5$ nm and $D = 15 \pm 2$ nm. Blue and red dashed lines correspond to the median and mean values of the distribution, respectively.

1.2 Scanning Electron Microscopy

Scanning electron microscopy (SEM) was conducted on AuNRs 750 to characterize their size distribution and dispersity. Samples were imaged at 20 kV with a dwell time of 30 μs. A representative SEM micrograph is shown in Fig. S2. The analysis revealed a homogeneous population of nanorods within the sample space. The size of these nanorods was found to be 15×52 nm as shown in Fig S3.

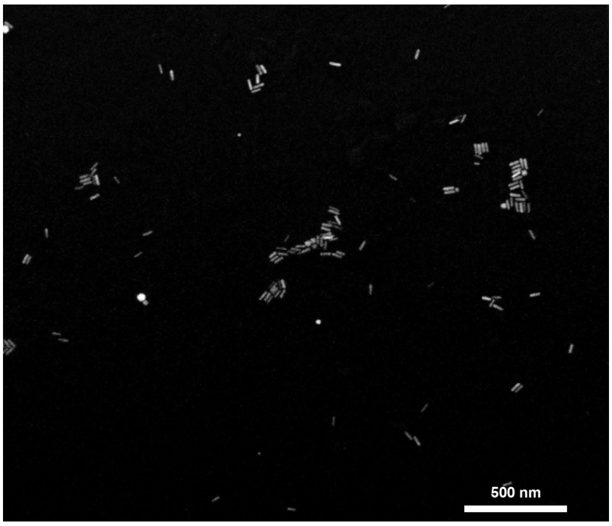

Figure S2: Representative SEM micrograph of sample AuNRs 750.

1.3 Depolarized DLS

The normalized VV and VH autocorrelation functions are shown in Fig. S4a and b, respectively. In the insets of the figures are the scaling of $g^{(2)}(\tau)^*$ with $q^2\tau$. The VV autocorrelation functions, $g^{(2)}_{VV}(\tau)$, exhibit a bimodal decay, which gradually diminishes to show a single relaxation mode as q increases. Although $g^{(2)}_{VV}(\tau)^*$ collapses on a single curve for several values of q, we note that this collapse is absent for

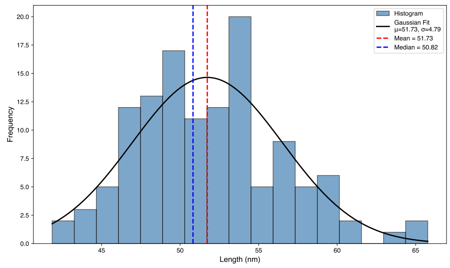

(a)

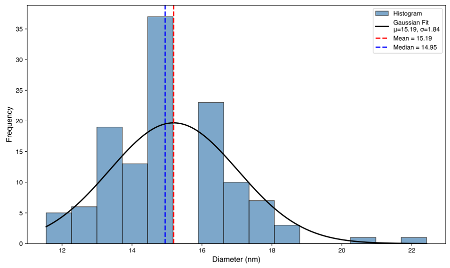

(b)

Figure S3: Histograms of Au nanorod (a) length and (b) diameter for sample AuNRs 750 obtained from SEM imaging. The black lines are fits to a Gaussian distribution, yielding average dimensions of $L = 52 \pm 5$ nm and $D = 15 \pm 3$ nm. Blue and red dashed lines correspond to the median and mean values of the distribution, respectively.

scattering angles below 70°. The first decay of the VV signal exhibits no scaling across any scattering angles, likely encompassing contributions from both the translation and rotation diffusion of the nanorods. In contrast, the second decay does scale with $q^2\tau$ for angles 70° through 140°, with curves converging over each other. In addition, for the lowest values of q, corresponding to the largest length scales, we do not observe a full decay of the autocorrelation function and fits to the curves, shown as solid lines in the figure, do not adequately describe the data for large values of τ. We observe a single decay mode in $g^{(2)}_{VH}(\tau)^*$, as expected from theory. The inset shows the scaling of $g^{(2)}_{VH}(\tau)^*$ with $q^2\tau$, showing a spread of scaled correlation functions as expected. Consequently, we only analyzed the autocorrelation data for angles 70° through 140° to determine the translational diffusion coefficient, D_{tr}. DDLS measurements from this restricted range of the VV signal are shown in Fig. S5.

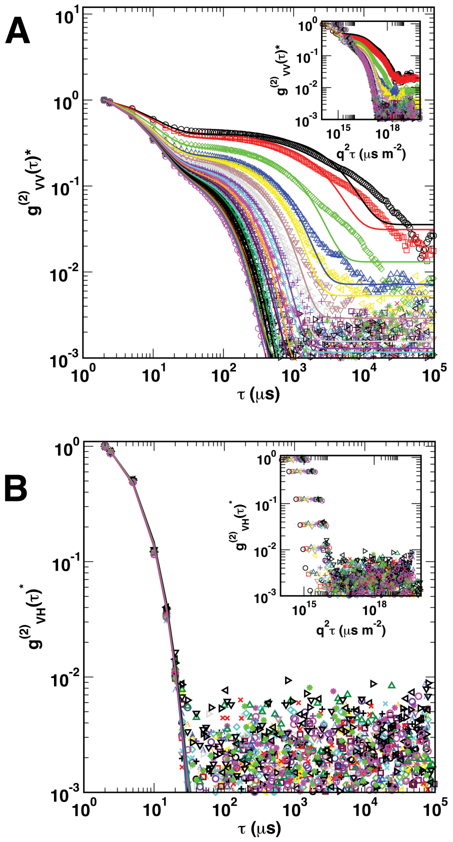

Figure S4: Representative DDLS measurements of AuNRs 750 as a function of scattering vector q from $q \approx 0.0068$ nm^{-1} to $q \approx 0.025$ nm^{-1}. The graphs show (a) the normalized autocorrelation function for the VV polarization, $g^{(2)}_{VV}(\tau)^*$, $f = 0.38 - 0.48$, and (b) the normalized autocorrelation function for the VH polarization condition, $g^{(2)}_{VH}(\tau)^*$, $f = 0.34 - 0.47$. In the main plot, curves from left to right correspond to decreasing values of q. Conversely, in the insets of both figures, which show the scaling of $g^{(2)}(\tau)^*$ with $q^2\tau$, the curves from left to right correspond to increasing values of q.

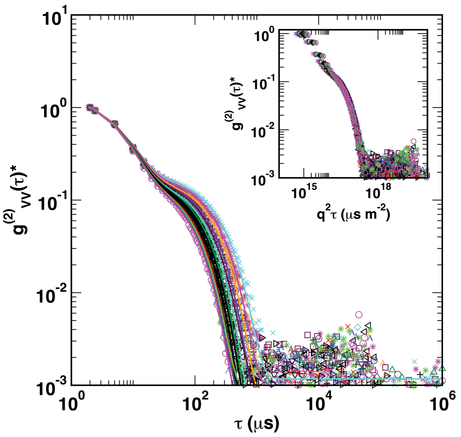

Figure S5: Vertically polarized normalized autocorrelation function taken from scattering angles of 70° to 140° for AuNRs 750 plotted against τ and $q^2\tau$ (inset). The data collapses when scaled by $q^2\tau$, suggesting the measurements capture purely translational motion.

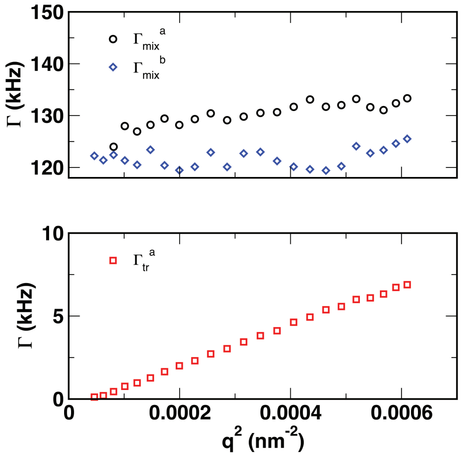

Figure S6: Relaxation rates Γ as the function of q^2 for AuNRs 750. In the panel, the top graph plots the mixed relaxation rate $\Gamma_{mix} = \Gamma_{tr} + \Gamma_{rot}$, while the bottom graph plots only the translational relaxation rate, Γ_{tr}. Γ^a_{mix} and Γ^b_{mix} refer to mixed relaxation rates taken from the fast VV mode and VH, respectively.

2 AuNRs 900 Data Analysis

2.1 Transmission Electron Microscopy

Transmission electron microscopy (TEM) analysis was conducted on AuNRs 900 to characterize their size distribution and dispersity. The analysis revealed a bimodal population of nanorods within the sample space. Representative TEM images are provided in the main text. The sizes of these two populations of nanorods were found to be 15×68 nm and 6×39 nm, as shown in Fig. S7.

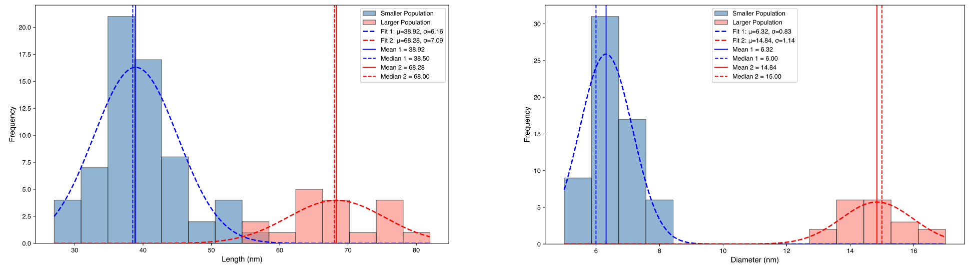

(a) (b)

Figure S7: Histograms of Au nanorod (a) length and (b) diameter for sample AuNRs 900 obtained from TEM imaging. The blue curves correspond to a population of nanorods with dimensions of 6×39 nm, whereas the red curves correspond to a population of nanorods with dimensions of 15×68 nm.

2.2 Scanning Electron Microscopy

Scanning electron microscopy (SEM) was conducted on AuNRs 900 to characterize their size distribution and dispersity. Samples were imaged at 20 kV with a dwell time of 30 μs. A representative SEM micrograph is shown in Fig. S8. The analysis revealed a homogeneous population of nanorods within the sample space, possibly due to the lower resolution of the SEM compared to the TEM. The sizes of this population of nanorods was 14×69 nm, as shown in Fig. S9.

2.3 Depolarized DLS

DDLS measurements for AuNRs 900 are shown in Fig. S10 for (a) the VV and (b) VH polarization states, with the scaling of the autocorrelation functions with $q^2\tau$ shown in the insets. In the main plot, curves from left to right correspond to decreasing values of q. The VV autocorrelation functions exhibits

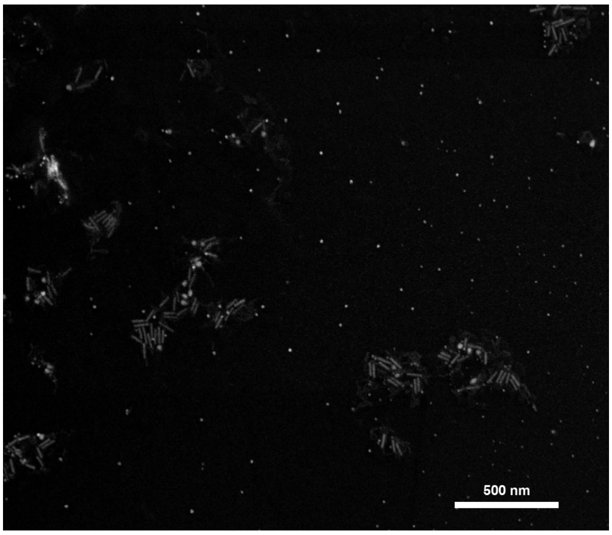

Figure S8: Representative SEM micrograph of sample AuNRs 900.

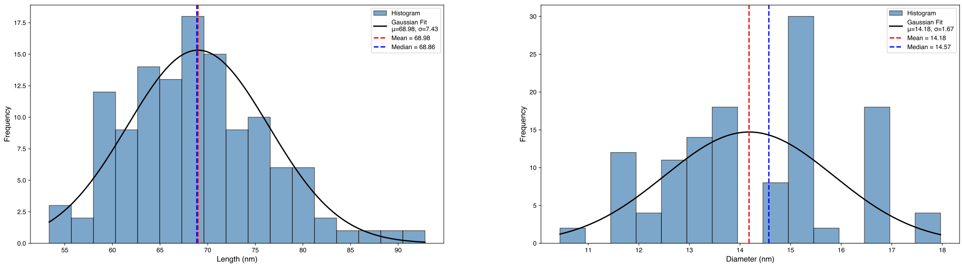

(a)

(b)

Figure S9: Histograms of Au nanorod (a) length and (b) diameter for sample AuNRs 900 obtained from SEM imaging. The black lines are fits to a Gaussian distribution, yielding average dimensions of $L = 69 \pm 7$ nm and $D = 14 \pm 2$ nm. Blue and red dashed lines correspond to the median and mean values of the distribution, respectively.

a bimodal decay, with the bimodal features gradually diminishing as the scattering angle increases to 140°. The inset shows the scaling of $g_{VV}^{(2)}(\tau)^*$ with $q^2\tau$, demonstrating collapse of the purely translational slow mode. The normalized VH autocorrelation functions align with all scattering angles and exhibit a monomodal decay. The inset shows the scaling of $g_{VH}^{(2)}(\tau)^*$ with $q^2\tau$, showing a spread of scaled correlation functions as expected. Consequently, all angles were utilized to calculate D_{tr} and D_{rot}.

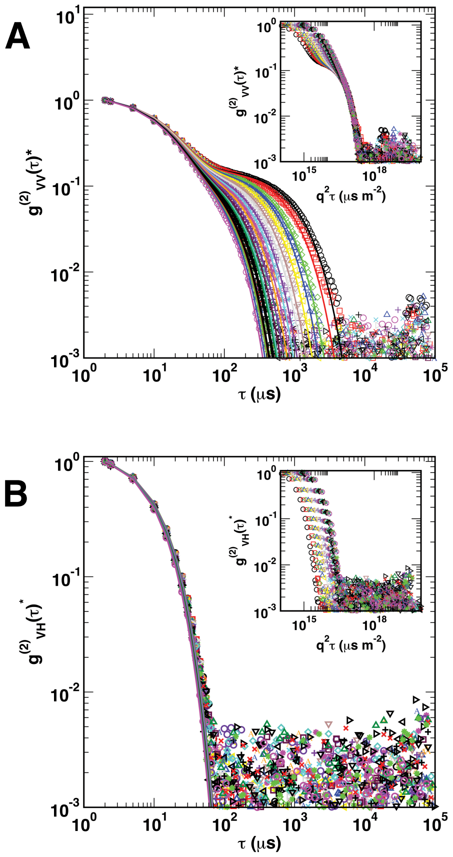

Figure S10: Representative DDLS measurements of AuNRs 900 as a function of scattering vector q from $q \approx 0.0068$ nm^{-1} to $q \approx 0.025$ nm^{-1}. The graphs show (a) the normalized autocorrelation function for the VV polarization, $g_{VV}^{(2)}(\tau)^*$, $f = 0.26 - 0.44$ and (b) the normalized autocorrelation function for the VH polarization, $g_{VH'}^{(2)}(\tau)^*$, $f = 0.21 - 0.30$. In the main plot, curves from left to right correspond to decreasing values of q. Conversely, in the insets of both figures, which show the scaling of $g^{(2)}(\tau)^*$ with $q^2\tau$, the curves from left to right correspond to increasing values of q.

3 AuNRs 980 Data Analysis

3.1 Transmission Electron Microscopy

Transmission electron microscopy (TEM) analysis was conducted on AuNRs 980 to characterize their size distribution and dispersity. Representative TEM images are provided in the main text. The analysis revealed a homogeneous population of nanorods within the sample space. The size of these nanorods was found to be 18 nm × 104 nm as shown in Fig. S11.

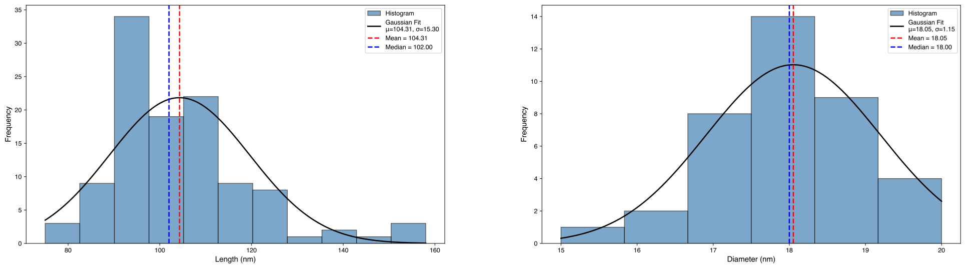

(a) (b)

Figure S11: Histograms of Au nanorod (a) length and (b) diameter for sample AuNRs 980 obtained from TEM imaging. The black lines are fits to a Gaussian distribution, yielding average dimensions of $L = 104 \pm 15$ nm and $D = 18 \pm 1$ nm. Blue and red dashed lines correspond to the median and mean values of the distribution, respectively.

3.2 Scanning Electron Microscopy

Scanning electron microscopy (SEM) was conducted on AuNRs 980 to characterize their size distribution and dispersity. Samples were imaged at 20 kV with a dwell time of 30 μs. A representative SEM micrograph is shown in Fig. S12. The analysis revealed a homogeneous population of nanorods. The size of these nanorods was found to be 17 nm × 100 nm as shown in Fig. S13.

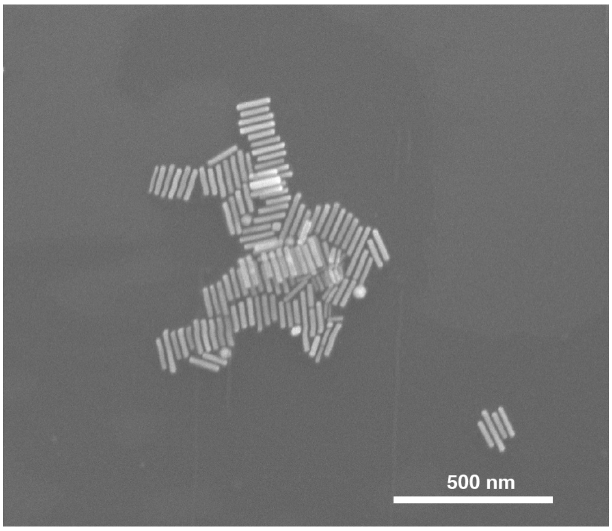

Figure S12: Representative SEM micrograph of sample AuNRs 980.

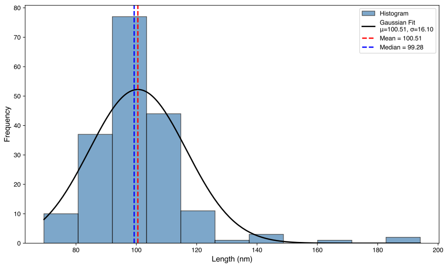

(a)

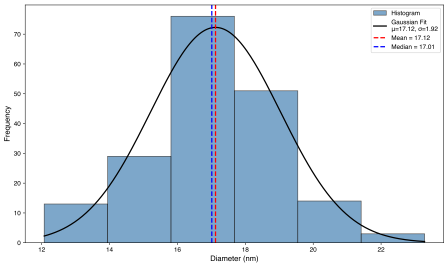

(b)

Figure S13: Histograms of Au nanorod (a) length and (b) diameter for sample AuNRs 980 obtained from SEM imaging. The black lines are fits to a Gaussian distribution, yielding average dimensions of $L = 100 \pm 16$ nm and $D = 17 \pm 2$ nm. Blue and red dashed lines correspond to the median and mean values of the distribution, respectively.

4 Depolarized DLS Analysis Neglecting Fast VV Mode

4.1 Approach 2: Aspect Ratio Guess

Table S1 summarizes the values of length and diameter obtained by neglecting the fast VV mode of $g^{(2)}_{VV}(\tau)^*$ for aspect ratio guess method. D_{tr} was calculated from the average slope of Γ^a_{tr} and Γ^b_{mix} plotted against q^2, whereas D_{rot} was determined from the y-intercept of Γ^b_{mix} (from the VH signal).

Sample	SC		PE		SP[a]	
	L (nm)	D (nm)	L (nm)	D (nm)	L (nm)	D (nm)
AuNRs 980	110	28	137	30	122	28
AuNRs 900	79	19	98	21	87	19
AuNRs 750	35	39	56	43	-	-

Table S1: Length (L) and diameter (D) of AuNRs samples obtained using Approach 2 without the fast VV mode for the straight cylinder (SC), prolate ellipsoid (PE), and spherocylinder (SC) models. [a]The SP model did not reach convergence for the AuNRs 750 sample.

5 Depolarized DLS Analysis Using Fast VV Mode

5.1 Approach 1: Solving for Aspect Ratio

The translational diffusion coefficients for the Au nanorods can be obtained from the VV or VH signal. The slow mode of the VV signal corresponds to translational motion such that $D_{tr} = q^{-2}\Gamma_{tr}$. In addition, both the decay of the VH signal and the fast mode of the VV signal are described by a relaxation rate that is a mixture of translational and rotational motions, i.e., $\Gamma_{mix} = \Gamma_{tr} + \Gamma_{rot}$. When Γ_{mix} is plotted against q^2, the slope of the curve provides D_{tr} while the y-intercept provides D_{rot}. Table S2 summarizes the values of D_{tr} and D_{rot} obtained by considering fast VV mode of $g^{(2)}_{VV}(\tau)^*$. D_{tr} was calculated from the average slope of Γ^a_{tr}, Γ^a_{mix}, and Γ^b_{mix} when plotted against q^2, while D_{rot} was determined from the average y-intercept of Γ^a_{mix} (from the VV signal) and Γ^b_{mix} (from the VH signal).

Sample	D_{tr} (cm^2/s)	D_{rot} (kHz)	SC		PE		SP	
			L (nm)	D (nm)	L (nm)	D (nm)	L (nm)	D (nm)
AuNRs 980	1.07×10^{-7}	3.10	147	8	163	9	147	9
AuNRs 900	1.39×10^{-7}	8.54	99	9	111	10	100	9
AuNRs 750	9.53×10^{-8}	20.51	35	43	53	48	44	63

Table S2: Translational (D_{tr}) and rotational (D_{rot}) diffusion coefficients, length and diameter predictions for AuNRs using Approach 1 with the fast VV mode included for the straight cylinder (SC), prolate ellipsoid (PE), and spherocylinder (SP) models.

5.2 Approach 2: Aspect Ratio Guess

Sample	SC		PE		SP[a]	
	L (nm)	D (nm)	L (nm)	D (nm)	L (nm)	D (nm)
AuNRs 980	148	8	163	9	147	9
AuNRs 900	99	9	111	10	100	9
AuNRs 750	34	41	53	50	-	-

Table S3: Length and diameter predictions for AuNRs using Approach 2 with the fast VV mode included for the straight cylinder (SC), prolate ellipsoid (PE), and spherocylinder (SP) models. [a]The SP model did not reach convergence for the AuNRs 750 sample.

5.3 Approach 3: Genetic Optimization

Sample	SC		PE		SP	
	L (nm)	D (nm)	L (nm)	D (nm)	L (nm)	D (nm)
AuNRs 980	104	30	132	30	117	30
AuNRs 900	78	20	99	18	87	18
AuNRs 750	42	28	64	30	53	30

Table S4: Length and diameter predictions for AuNRs using Approach 3 with the fast VV mode included for the straight cylinder (SC), prolate ellipsoid (PE), and spherocylinder (SP) models.

6 DDLS Mode Amplitudes

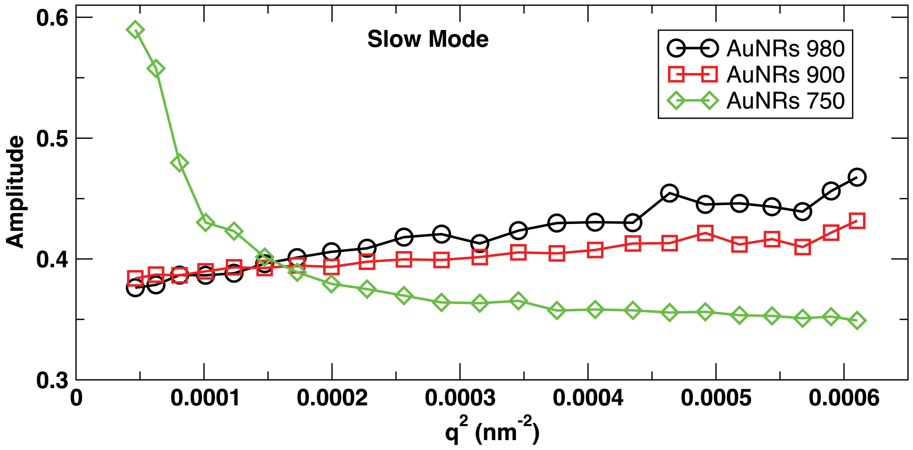

Figure S14: Relative amplitude of the slow decay mode A_{slow} extracted from bimodal fits to the VV intensity autocorrelation functions, plotted as a function of q^2 for AuNRs 980 (black circles), AuNRs 900 (red squares), and AuNRs 750 (green diamonds). The amplitude of the fast mode (A_{fast}) is determined by the constraint $A_{fast} = 1 - A_{slow}$.

7 Calculation of the Depolarization Ratio (Δ)

7.1 Discrete Dipole Approximation

The depolarization ratio describes the relative intensity of the VH scattering intensity relative to the VV scattering intensity: $\Delta = I_{VH}/I_{VV}$. The depolarization ratio was determined from Discrete Dipole Approximation (DDA) calculations of the Mueller matrix, $\mathbf{M}$. DDA calculations were performed using DDSCAT 7.3,[1] compiled with double precision accuracy and OpenMP support. Wavelength-dependent refractive index values for Au were obtained from Weaver[2], and corrected for surface damping from collisions of the electrons with the Au nanorod surface.[3] We computed the scattering from a single, spherocylindrical nanorod embedded in a medium with constant refractive index $n = 1.00$. The number of dipoles in the Au nanorod was chosen such that the lattice dispersion relation $|m|kd < 0.01$, where $|m|$ is the complex refractive index of Au, k is the wavenumber, and d is the effective size of the target. $\mathbf{M}$ was obtained from an orientational average of the nanorod with respect to the incident light. A more detailed discussion of DDA and its application to modeling Au nanorods can be found in the literature.[4] To determine the effect of surface plasmon resonances in AuNRs on light scattering, we compared two AuNRs with diameters of 15 nm, and lengths of 50 nm and 105 nm to Au nanospheres (diameter = 30 nm) and a cellulose nanorod (15 nm x 50 nm). Cellulose refractive index data were taken from Niskanen et al.[5]

7.2 Calculation of VV and VH Scattering Intensity

To calculate Δ, incident light was described by a Stokes vector

$$\mathbf{S}_0 = I_0 \begin{pmatrix} 1 \\ -1 \\ 0 \\ 0 \end{pmatrix} \tag{1}$$

where I_0 is the intensity of the light. $\mathbf{S}_0$ corresponds to linearly polarized light, with the electric field perpendicular to the scattering plane. After interaction with the target, the scattered light is described by a new Stokes vector:

$$\mathbf{S}_s = \frac{1}{k^2r^2}\mathbf{M}\mathbf{S}_0 = \frac{I_0}{k^2r^2}\begin{pmatrix} S_{11} & S_{12} & S_{13} & S_{14} \\ S_{21} & S_{22} & S_{23} & S_{24} \\ S_{31} & S_{32} & S_{33} & S_{34} \\ S_{41} & S_{42} & S_{43} & S_{44} \end{pmatrix}\begin{pmatrix} 1 \\ -1 \\ 0 \\ 0 \end{pmatrix} = \frac{I_0}{k^2r^2}\begin{pmatrix} S_{11} - S_{12} \\ S_{21} - S_{22} \\ S_{31} - S_{32} \\ S_{41} - S_{42} \end{pmatrix} \tag{2}$$

where k is the wavenumber and r is the distance from the target. The effect of the analyzer on the DDLS instrument is accounted for by applying a second Mueller matrix describing an ideal linear polarizer:

$$\mathbf{M}_p(\xi) = \frac{1}{2}\begin{pmatrix} 1 & \cos 2\xi & \sin 2\xi & 0 \\ \cos 2\xi & \cos^2 2\xi & \cos 2\xi \sin 2\xi & 0 \\ \sin 2\xi & \sin 2\xi \cos 2\xi & \sin^2 2\xi & 0 \\ 0 & 0 & 0 & 0 \end{pmatrix} \tag{3}$$

where the polarizer orientation $\xi = 0$ in the VV condition, and $\xi = \pi/2$ in the VH condition. Application of the polarizer yields (for the intensity components of the Stokes vector):

$$I_{VV} = \mathbf{M}_p(0)\mathbf{S}_s = \frac{I_0}{2k^2r^2}\left(S_{11} - S_{12} + S_{21} - S_{22}\right) \tag{4}$$

and

$$I_{VH} = \mathbf{M}_p\left(\frac{\pi}{2}\right)\mathbf{S}_s = \frac{I_0}{2k^2r^2}\left(S_{11} - S_{12} - S_{21} + S_{22}\right) \tag{5}$$

From these scattered intensities, the depolarization ratio is computed as:

$$\Delta = \frac{I_{VH}}{I_{VV}} = \frac{S_{11} - S_{12} - S_{21} + S_{22}}{S_{11} - S_{12} + S_{21} - S_{22}} \tag{6}$$

Shown in Fig. S15 is Δ plotted as a function of laser wavelength for $\lambda = 400$ nm to $\lambda = 700$ nm comparing the response of Au nanorods (red and blue lines) with Au nanospheres (black) and cellulose nanorods (green). Both the Au nanosphere and cellulose nanorod show $\Delta = 0$ across the entire wavelength range, suggesting that no VH signal would be observed experimentally. For the two Au nanorods, a maximum in Δ is observed near $\lambda = 600$ to 650 nm, depending on the dimensions of the nanorod. Experimental measurements of Δ by Fytas et al. of Δ for AuNRs showed very similar trends.[6] Cellulose does not produce surface plasmons, implying that the behavior of Δ that we observe in these calculations originates from the optical response of Au.

As noted by Fytas and coworkers,[6] the VV signal contains isotropic contributions (i.e., the slow VV mode) as well as contributions from the VH signal. If Δ is small, the VV signal will be dominated by the slow mode. However, as shown in Fig. S15, our nanorods have large values of Δ near $\lambda = 637$ nm suggesting that the VH signal will contribute strongly to the VV measurement, as we observed. We attribute the fast VV mode to this effect. To support this conclusion, we performed DDLS measurements on AuNRs 980 using a 488 nm laser. As shown in Fig. S15, $\Delta < 0.1$ for $\lambda = 488$ nm, and the VH signal is expected to be weak. Similarly, one would not expect to see a fast VV mode. Figure S16a shows the VV signal from the measurement with the 488 nm laser, demonstrating the lack of a fast mode. As shown in Fig. S16b, the VH signal is weak and contains significant noise as a result. Therefore, we conclude that the surface plasmons in the AuNRs and their effect on the depolarization ratio lead to the fast VV mode that was observed at $\lambda = 637$ nm.

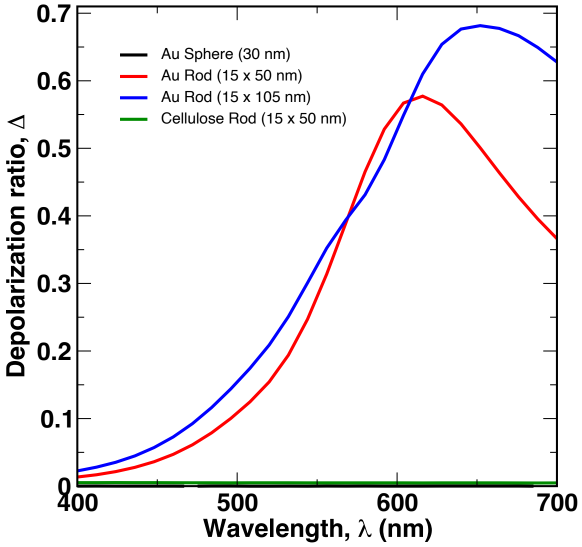

Figure S15: Depolarization ratio Δ plotted as a function of wavelength for a Au nanosphere (30 nm, black), Au nanorod (15×50 nm, red), Au nanorod (15×105 nm, blue), and cellulose nanorod (15×50 nm, green). The cellulose nanorod and Au nanosphere show a constant $\Delta = 0$.

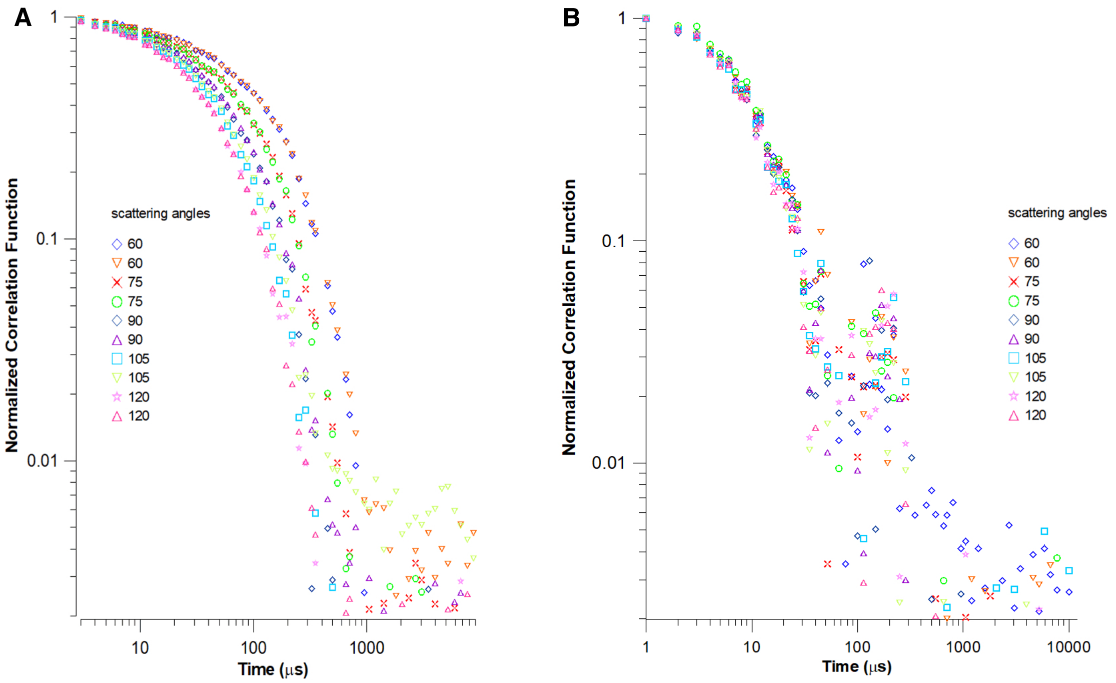

Figure S16: DDLS measurements made at $\lambda = 488$ nm for sample AuNRs 980. (a) The lack of a fast mode in the VV measurement is attributed to a low value of the depolarization ratio for the nanorods at this wavelength, as shown in Fig. S15. (b) The VH measurement shows weak scattering due to the low value of Δ.

References

[1] B. T. Draine and P. J. Flatau, *Journal of the Optical Society of America A*, 1994, **11**, 1491–1499.

[2] J. H. Weaver, C. Krafka, D. Lynch and E. Koch, *Applied optics*, 1981, **20**, 1124_1–1125.

[3] M. J. Hore, A. L. Frischknecht and R. J. Composto, *ACS Macro Letters*, 2012, **1**, 115–121.

[4] M. J. Hore, in *Theory and Modeling of Polymer Nanocomposites*, Springer, 2020, pp. 259–280.

[5] I. Niskanen, T. Suopajärvi, H. Liimatainen, T. Fabritius, R. Heikkilä and G. Thungström, *Journal of Quantitative Spectroscopy and Radiative Transfer*, 2019, **235**, 1–6.

[6] M. Haghighi, M. A. Plum, G. Gantzounis, H.-J. Butt, W. Steffen and G. Fytas, *The Journal of Physical Chemistry C*, 2013, **117**, 8411–8419.
